# STX13 regulates cargo delivery from recycling endosomes during melanosome biogenesis

**DOI:** 10.1242/jcs.171165

**Published:** 2015-09-01

**Authors:** Riddhi Atul Jani, Latha Kallur Purushothaman, Shikha Rani, Ptissam Bergam, Subba Rao Gangi Setty

**Affiliations:** 1 Department of Microbiology and Cell Biology, Indian Institute of Science, Bangalore 560 012, India; 2 Institut Curie, Centre de Recherche, Paris 75248, France; 3 Structure and Membrane Compartments, and Cell and Tissue Imaging Facility (PICT-IBiSA), CNRS UMR144, Paris 75248, France

**Keywords:** STX13, VAMP7, SNARE recycling, TYRP1, TYR, AP-3, HPS

## Abstract

Melanosomes are a class of lysosome-related organelles produced by melanocytes. Biogenesis of melanosomes requires the transport of melanin-synthesizing enzymes from tubular recycling endosomes to maturing melanosomes. The SNARE proteins involved in these transport or fusion steps have been poorly studied. We found that depletion of syntaxin 13 (STX13, also known as STX12), a recycling endosomal Qa-SNARE, inhibits pigment granule maturation in melanocytes by rerouting the melanosomal proteins such as TYR and TYRP1 to lysosomes. Furthermore, live-cell imaging and electron microscopy studies showed that STX13 co-distributed with melanosomal cargo in the tubular-vesicular endosomes that are closely associated with the maturing melanosomes. STX family proteins contain an N-terminal regulatory domain, and deletion of this domain in STX13 increases both the SNARE activity *in vivo* and melanosome cargo transport and pigmentation, suggesting that STX13 acts as a fusion SNARE in melanosomal trafficking pathways. In addition, STX13-dependent cargo transport requires the melanosomal R-SNARE VAMP7, and its silencing blocks the melanosome maturation, reflecting a defect in endosome–melanosome fusion. Moreover, we show mutual dependency between STX13 and VAMP7 in regulating their localization for efficient cargo delivery to melanosomes.

## Abstract (precis)

**Highlighted Article:** The SNAREs STX13 and VAMP7 mutually regulate their localization in melanocytes and control the cargo delivery to the melanosome during its biogenesis.

## INTRODUCTION

Melanin pigments are synthesized in melanosomes, a melanocyte-specific lysosome-related organelle (LRO) that coexists with conventional lysosomes. These organelles originate from the endocytic system and play a key role in skin color and photoprotection against ionizing radiation (Dell'Angelica, 2004; Raposo et al., 2007). Melanosome biogenesis requires efficient and accurate transport of melanin-synthesizing enzymes from tubular or vesicular recycling endosomes to pre-mature melanosomes, which then undergo further maturation into fully pigmented melanosomes (Marks et al., 2013; Sitaram and Marks, 2012). Mutations in certain genes that encode multi-subunits protein complexes such as the biogenesis of lysosome-related organelles complexes (BLOC-1, BLOC-2 and BLOC-3) and the adaptor protein (AP)-3 complex, disrupt the cargo transport to melanosomes and other LROs, resulting in Hermansky–Pudlak syndrome (HPS), which is characterized by oculocutaneous albinism, prolonged bleeding and other symptoms (Di Pietro and Dell'Angelica, 2005; Huizing et al., 2008; Wei, 2006). Moreover, Rabs (Rab7, Rab38 and Rab32), adaptors (AP-1), cytoskeleton regulators (the WASH complex), motor proteins (KIF13A) and Rab effectors (VARP, also known as ANKRD27) also regulate the melanosomal cargo delivery either from recycling endosomal domains or the Golgi in association with HPS protein complexes (Bultema et al., 2012; Delevoye et al., 2009; Gerondopoulos et al., 2012; Hida et al., 2011; Ryder et al., 2013; Tamura et al., 2011; Wasmeier et al., 2006). Previous studies have suggested that the transport of melanosomal proteins to melanosomes occurs in two different routes from recycling endosomes: (1) through BLOC-1-dependent transport of tyrosinase-related protein-1 (TYRP1) and other cargo, and (2) through AP-3-dependent transport of tyrosinase (TYR) to maturing melanosomes containing premelanosomal protein (PMEL) fibrils (Marks et al., 2013; Sitaram and Marks, 2012). Additionally, electron tomographic analysis of melanocytes has visualized the direct contact of recycling endosome domains harboring melanosomal cargo with the limiting membrane of melanosomes (Delevoye et al., 2009). However, the SNARE proteins involved in such fusion events remain unidentified.

The SNARE family contains at least 38 proteins in human, which are classified as either ‘Q’ or ‘R’ SNAREs, and is known to control all membrane fusion events (Hong, 2005; Jahn and Scheller, 2006). It has been observed that the expression of multiple SNAREs varied with melanogenesis during the differentiation of B16 melanoma cells (Wade et al., 2001), but their role in melanosome biogenesis has never been demonstrated. Studies have also shown that the depletion of STX3 (a Qa-SNARE) or vesicle-associated membrane protein 7 (VAMP7; an R-SNARE) in melanocytes affects the transport of TYRP1 to melanosomes (Tamura et al., 2011; Yatsu et al., 2013). Nevertheless, the function of other SNAREs still remains unknown considering a variety of cargo molecules need to be transported for melanogenesis. In our previous studies, we have shown that, in BLOC-1-deficient melanocytes, melanosomal proteins mislocalize to STX13 (a Qa-SNARE)-positive endosomal structures that are enriched in recycling endosome tubular structures (Setty et al., 2008, 2007). Furthermore, pallidin (also known as BLOC1S6), a subunit of BLOC-1 has been shown to interact with STX13 in a yeast two-hybrid assay (Huang et al., 1999; Moriyama and Bonifacino, 2002). However, none of these studies have clearly illustrated or provided evidence for the function of STX13 as a SNARE in cargo transport or melanosome biogenesis pathways. Studies have shown that STX13 regulates the recycling of cargo from endosomal tubular extensions to the plasma membrane (Kean et al., 2009; Prekeris et al., 1998; Williams and Coppolino, 2014) in addition to its role in homotypic endosome–endosome fusion (McBride et al., 1999; Sun et al., 2003) and in autophagosome maturation (Lu et al., 2013). Although, STX13 has been shown to mostly function at the plasma membrane, its role in endosomal cargo transport to an LRO, such as melanosomes, has never been studied. Additionally, the endosomal distribution of STX13 in melanocytes is dependent on AP-3 complex (Setty et al., 2007), but the role of this SNARE-adaptor regulation in melanosome pigmentation has not been addressed.

In this study, we set out to determine the function of STX13 in regulating the cargo delivery steps to melanosomes. Here, we employ immortalized mouse melanocytes derived from HPS models, mutational analysis and knockdown of SNAREs, studied localization and trafficking of melanosomal proteins, including SNAREs, and used live-cell imaging of endosomal tubular intermediates to place STX13, as well as VAMP7, in the melanosome biogenesis pathway. Thus, our data demonstrate that STX13 regulates melanosomal cargo transport and biogenesis through its cycling between recycling endosomal domains and melanosomes in a mutual regulation with VAMP7.

## RESULTS

### STX13 is required for melanocyte pigmentation

**Fig. 1.**
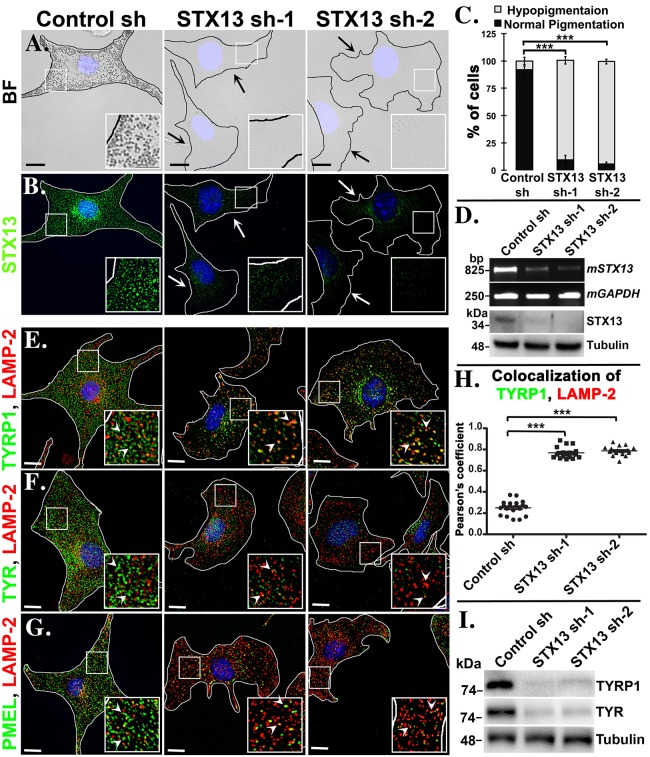
**STX13 knockdown affects the melanocyte pigmentation and cargo transport.** (A,B,E–G), Bright-field (BF) microscopy and immunofluorescence microscopy images of STX13-depeleted and control melanocytes. Arrows indicate the loss in pigmentation (A) or STX13 staining (B), and arrowheads (E–G) point to the localization of melanosome cargo to lysosomes in knockdown cells. Nuclei are stained with Hoechst 33258. The insets are a magnified view of the white boxed areas. Scale bars, 10 μm. (C,D,H,I) Visual quantification of melanocyte pigmentation (*n*=3, ≥100 cells/sample, ***P<0.001, mean±s.e.m.) (C), semiquantitative PCR (top) and immunoblotting analysis (bottom) (D), degree of colocalization between TYRP1 and LAMP-2, ***P<0.001, mean±s.e.m. (H), and immunoblotting analysis of melanosomal proteins (I) in STX13-knockdown cells.

Melanosomes acquire melanin biosynthetic enzymes through recycling endosomal fusion events (Delevoye et al., 2009; Setty et al., 2007; Theos et al., 2005). STX13 has been shown to localize to the recycling endosomes (Prekeris et al., 1998), but its role in cargo transport to melanosomes or melanocyte pigmentation remains unknown. Knockdown of STX13 using two independent short hairpin RNAs (shRNAs, denoted STX13 sh-1 and sh-2) significantly reduced the pigmentation of wild-type (melan-Ink4a) melanocytes (Fig. 1A), suggesting a defect in melanosome biogenesis and/or maturation. Visual quantification of cellular pigmentation by bright-field microscopy provided evidence that almost 90% of melanocytes were hypopigmented upon STX13 knockdown (Fig. 1C, percentage of hypopigmented cells = 8.02±3.39 in control sh, 90.90±3.62 in STX13 sh-1 and 93.95±2.23 in STX13 sh-2, *n*=3; mean±s.e.m.). Correspondingly, the endogenous level of STX13 was reduced in STX13-depleted melanocytes as observed by immunofluorescence microscopy (Fig. 1B), which is consistent with reduced expression of both transcript and protein levels in the cells (Fig. 1D). Importantly, expression of human GFP–STX13 in STX13-depleted cells resulted in partial rescue of melanocyte pigmentation (supplementary material Fig. S1A). However, the localization of endosomal proteins such as Rab5 and adaptors, AP-1 or AP-3 were not affected upon STX13 knockdown, consistent with the absence of any change in AP-1 (γ) or AP-3 (σ) subunit expression (supplementary material Fig. S1B,C), indicating that the identity and distribution of different endosomal domains is unaffected in the STX13-depleted melanocytes. Thus, these findings suggest a specific role for STX13 in melanosome pigmentation and biogenesis.

### STX13 regulates the cargo delivery by shuttling between endosomes and melanosomes

Given that STX13 knockdown affects melanocyte pigmentation, we examined whether STX13 is required for cargo transport to the melanosomes. We analyzed the steady-state distribution of three primary melanosomal proteins, namely TYRP1 (BLOC-1-dependent), TYR (AP-3-dependent) and PMEL (BLOC-1- and AP-3-independent) with respect to the lysosomal protein LAMP-2 in STX13-knockdown cells by immunofluorescence microscopy. Unlike the control cells, STX13-depleted cells showed accumulation of TYRP1 in LAMP-2-positive structures (Fig. 1E, quantified in Fig. 1H; Pearson's coefficient, *r*=0.25±0.02 in control, 0.77±0.01 in STX13 sh-1 and 0.79±0.01 in STX13 sh-2; mean±s.e.m.), indicating that the bulk of TYRP1 is misrouted to lysosomes upon STX13 depletion. Consistent with this, TYRP1 expression was reduced in STX13-knockdown cells (Fig. 1I) and restored upon the ectopic expression of GFP–STX13 (supplementary material Fig. S1A) or treatment with bafilomycin A1 (supplementary material Fig. S1E). Interestingly, inactivation of STX13 significantly reduced the expression of TYR, a BLOC-1 independent cargo (Fig. 1I). Accordingly, the TYR activity, as judged using the substrate L-DOPA, was reduced dramatically in the STX13-knockdown cells even in the presence of 20 μM copper (a cofactor required for TYR activity) (Setty et al., 2008), suggesting that TYR expression was destabilized in the cells (supplementary material Fig. S1D). Furthermore, the low steady-state levels of stained TYR in STX13-depleted cells appeared as punctate structures with no clear colocalization with the lysosomes (Fig. 1F). However, the TYR expression to LAMP-2-positive lysosomes was restored with bafilomycin A1 (supplementary material Fig. S1F), indicating that TYR is misrouted and quickly degraded in the lysosomes upon STX13 depletion. Moreover, the observed residual TYR expression (Fig. 1F) very likely represented a biosynthetic pool in the Golgi that is still associated with a basal level of TYR activity in STX13-knockdown cells (arrowheads in supplementary material Fig. S1D). Immature or pre-melanosomes (stage II) are characterized by the presence of PMEL-positive fibers (Berson et al., 2001). Surprisingly, we found that the expression of PMEL was substantially reduced and targeted to lysosomes in STX13-knockdown cells (Fig. 1G), suggesting a defect in transport or biogenesis of PMEL-positive compartments. Thus, these results suggest that melanosomal proteins are targeted to lysosomes for degradation in the STX13-depleted melanocytes.

**Fig. 2.**
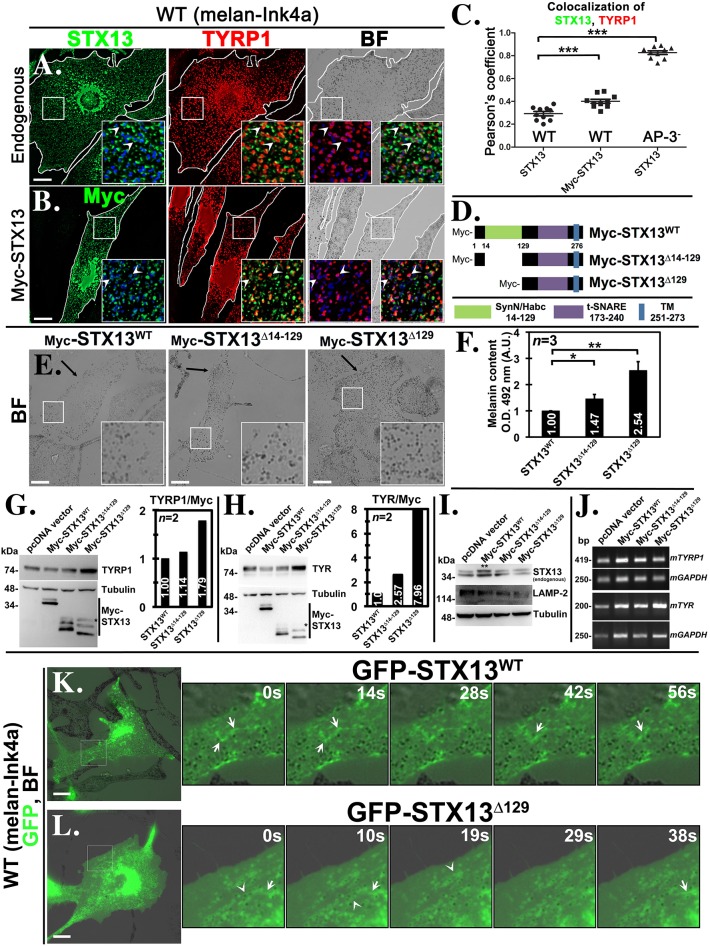
**Regulatory-domain-deficient STX13 increases the melanocyte pigmentation and cargo delivery upon overexpression.** (A,B) Bright-field (BF) microscopy and immunofluorescence microscopy images of untransfected (A) or Myc–STX13-transfected (B) melanocytes. Arrowheads point to the STX13 localization. (C) Degree of colocalization between endogenous or Myc–STX13 and TYRP1 in wild-type, wild-type expressing Myc–STX13 and AP-3^−^ melanocytes (*n*=10 cells). ***P<0.001, mean±s.e.m. (D) Schematic representation of conserved domains and deletion mutations in STX13. (E–J) Bright-field microscopy (E), melanin estimation *P<0.05, **P<0.01, mean±s.e.m. (F), immunoblotting (G–I) and semiquantitative PCR (J) of melanocytes expressing different Myc–STX13 mutants. Arrows point to the melanocyte pigmentation in E. ‘*’ indicates the proteolyzed band of Myc–STX13 (G,H) and ‘**’ indicates both the endogenous and ectopic copy of STX13 (I). The graphs in G and H represent the fold change in expression of melanosomal proteins with respect to Myc–STX13 overexpression. Note that the protein band intensities of TYRP1, TYR and Myc–STX13 were normalized to the respective tubulin expression and then plotted as the ratio between TYRP1 or TYR with Myc–STX13 expression. (K,L) Time-lapse (GFP and bright-field) live imaging microscopy of melanocytes expressing GFP–STX13^WT^ (K) or GFP–STX13^Δ129^ (L). Arrows and arrowheads represent the tubular structures and melanosome localized STX13, respectively. The insets are a magnified view of the white boxed areas. Scale bars: 10 μm.

Next, we tested whether the STX13 cycles between endosomes and melanosomes for cargo delivery in wild-type melanocytes. Endogenous STX13 in melanocytes localizes predominantly to recycling tubular endosomes (Dennis et al., 2015) and these structures sometime are associated with melanosomes or partially colocalized with melanosomal protein TYRP1 (Fig. 2A, quantified in 2C; *r=*0.29±0.02, mean±s.e.m.). Interestingly, STX13 localization to melanosomes or colocalization with TYRP1 significantly increased upon overexpression (Myc–STX13) in addition to tubular endosomal domains in wild-type melanocytes (Fig. 2B, quantified in Fig. 2C; *r=*0.4±0.02, mean±s.e.m.). Furthermore, localization of STX13 to melanosomes has been shown to increase dramatically in AP-3-deficient melan-pe (deficient for the β3A subunit) (Setty et al., 2007) and melan-mh (deficient for the δ subunit) melanocytes (see Fig. 4A, quantified in Fig. 2C; *r=*0.83±0.02, mean±s.e.m.), indicating that STX13 partitions between endosomes and melanosomes in an AP-3-dependent manner. Moreover, live imaging microscopy showed that endosomal tubular structures containing GFP–STX13 were associated with melanosomes in wild-type melanocytes (Fig. 2K; supplementary material Movie 1). We have previously shown that these tubular structures were longer than 1 μm and their contact with melanosomes persisted from a few seconds up to a minute (Dennis et al., 2015). Furthermore, STX13-positive tubular structures are derived from early-endosome-positive Rab5 compartments and colocalize with recycling endosomal marker Rab11 (Dennis et al., 2015). These studies suggest that STX13 trafficking occurs from recycling endosomes to melanosomes and transiently localized to melanosomes upon overexpression. However, melanosome pigmentation or localization of melanosomal proteins such as TYRP1 was not significantly affected in Myc–STX13-transfected cells (Fig. 2B). These results indicate that STX13 is likely regulated by an intracellular machinery to control the membrane fusion with melanosomes. Overall, these studies demonstrate that endosomal STX13 cycles between recycling endosomes and melanosomes and also regulates the cargo trafficking to melanosomes.

### Regulatory-domain-deleted STX13 mutants increases the pigmentation by localizing to melanosomes

SNAREs of the syntaxin family possess an N-terminal unstructured regulatory domain, namely SynN or Habc domain, which regulates the SNAREpin (i.e. pairing of Qa, Qb and Qc SNAREs with an R-SNARE) formation by interacting with Sec1–Munc18 family proteins (Hong, 2005; Jahn and Scheller, 2006; Rizo, 2012). Further, SNAREs lacking this Habc domain (active SNARE) can still actively participate in SNARE complex formation and increase the membrane fusion events *in vitro* (Shen et al., 2010). Sequence analysis revealed that STX13 contains a regulatory domain at amino acids 14–129, followed by a t-SNARE domain (amino acids 173–240) and a transmembrane domain (amino acids 251–273) (Fig. 2D). We performed deletion mutagenesis in the N-terminus of Myc–STX13 and constructed Δ14–129 (deleted Habc domain only) and Δ129 mutants (Fig. 2D). Furthermore, we tested whether the Habc domain of STX13 regulates the SNARE activity *in vivo* and causes any gain in pigmentation or cargo delivery to melanosomes. Expression of STX13 mutants in wild-type melanocytes caused an increase in pigmentation and melanin content (Fig. 2E, quantified in Fig. 2F, 1.47±0.17 times in STX13^Δ14-129^ and 2.54±0.32 times in STX13^Δ129^, *n*=3, mean±s.e.m.) as compared to wild-type STX13 (STX13^WT^; Fig. 2E,F) or empty vector (data not shown). Correspondingly, the protein levels but not transcript levels of melanin biosynthetic enzymes, such as TYRP1 and TYR, were increased substantially in cells expressing STX13 mutants compared to STX13^WT^ or control melanocytes (Fig. 2G,H, shows the ratio of TYRP1 or TYR protein levels with Myc–STX13 expression normalized to the respective tubulin level, *n*=2; Fig. 2J, supplementary material Fig. S2A, shows the ratio of the transcripts TYRP1 or TYR with Myc–STX13 normalized to the respective GAPDH expression, *n*=2). In addition, protein levels of endogenous STX13 and LAMP-2 were not affected in these cells (Fig. 2I). These results indicate that STX13 mutants possess higher SNARE activity than STX13^WT^.

**Fig. 3.**
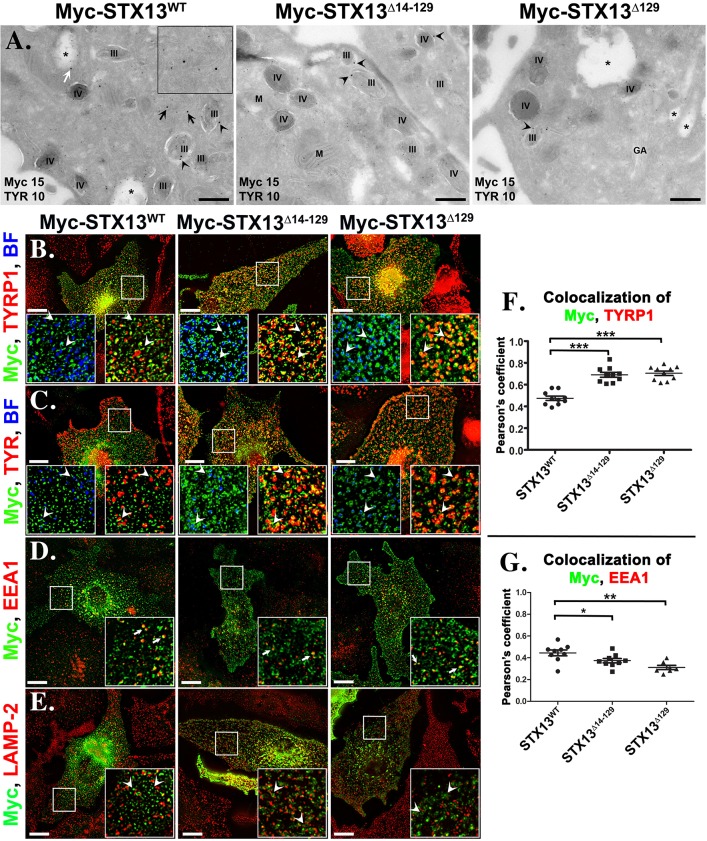
**Regulatory-domain-deficient STX13 mutants localizes to melanosomes.** (A) Immunoelectron microscopy images of stably expressing Myc–STX13^WT^ or mutant melanocytes. Myc (PAG15) and TYR (PAG10) represent the immunogold labeling of proteins with respective antibodies. Black and white arrows indicate the localization of STX13^WT^ to tubular and vacuolar endosomal domains, respectively. Arrowheads point to STX13 localization in melanosomes (stages III or IV, as indicated). Inset, an image emphasizing STX13- and/or melanosome cargo-positive tubular structures. ‘*’ vacuolar early endosomes; M, mitochondria; GA, Golgi. Scale bars: 400 nm. (B–E) Immunofluorescence microscopy images of melanocytes expressing Myc–STX13^WT^ or mutants. The bright-field (BF) image is pseudocolored blue. Arrowheads point to Myc–STX13 localization and arrows indicate the colocalization of Myc–STX13 with EEA1 in D. The insets are a magnified view of the white boxed areas. Scale bars: 10 μm. (F,G) Degree of colocalization between STX13 and TYRP1 (F) or EEA1 (G) (*n*=7–10 cells). *P<0.05, **P<0.01 and ***P<0.001; mean±s.e.m.

Live-cell imaging and electron microscopic studies have shown that delivery of melanosome cargoes such as TYRP1 occurs through endosome-derived tubular structures, which correspond to recycling endosomes (Delevoye et al., 2009; Dennis et al., 2015). We assessed the possibility that these domains also contain STX13^WT^ (see Fig. 3) and form similar tubular structures by using live imaging microscopy. GFP–STX13^WT^ in wild-type melanocytes showed few endosomal tubular structures that are associated with melanosomes (arrows in Fig. 2K; supplementary material Movie 1). In contrast, GFP–STX13^Δ129^ localized to melanosomes (see below) and occasionally appeared as either short tubular structures in the peripheral cytosol or longer tubular structures near the Golgi region (arrows in Fig. 2L; supplementary material Movie 2), indicating that STX13^Δ129^ also participates actively in cargo transport to melanosomes by localizing to the tubular endosomal domains. Furthermore, we tested whether another melanosomal protein, TYR, also localizes to these STX13-positive tubules. Time-lapse live-cell imaging showed that a subset of GFP–TYR colocalized with RFP–STX13-positive punctate or tubular structures in wild-type melanocytes (arrows, supplementary material Fig. S2B). Moreover, we analyzed the stable melanocytes expressing Myc–STX13 by immunoelectron microscopy on ultrathin cryosections immunogold-labeled for the Myc epitope and the melanosomal proteins TYR or TYRP1. Immunoelectron microscopy analysis showed that Myc–STX13^WT^ localized predominantly to tubular (black arrows and insets) and vacuolar domains (white arrow, star) of likely the endosomal structures closely associated to TYR or TYRP1-positive pigmented melanosomes (stage III and IV) (Fig. 3A; supplementary material Fig. S2C), consistent with the localization of GFP–STX13 in MNT1 cells (Dennis et al., 2015). In addition, a fraction of the TYR- or TYRP1-labeled tubular structures was positive for STX13^WT^ (black arrows and inset in Fig. 3A) (Dennis et al., 2015). Interestingly, STX13^WT^ was also associated with the limiting membrane of pigmented melanosomes (black arrowheads, Fig. 3A), suggesting that STX13-positive tubules might fuse with pigmented melanosomes. These studies suggest that STX13 mediates the trafficking of both TYR and TYRP1 (see below) to melanosomes by localizing to their respective endosomal tubular domains.

We examined whether Habc domain regulates STX13 trafficking in and out of melanosomes. In immunoelectron microscopy images, Habc-domain-deficient STX13 mutants (STX13^Δ14–129^ and STX13^Δ129^) were mostly co-distributed to either TYR- or TYRP1-positive limiting membranes of melanosomes (black arrowheads, Fig. 3A; supplementary material Fig. S2C). Furthermore, STX13 mutants were poorly associated to tubular or vacuolar (marked as star) endosomal domains compared to STX13^WT^ (Fig. 3A; supplementary material Fig. S2C). Consistent with immunoelectron microscopy data, immunofluorescence microscopy studies in wild-type melanocytes showed both STX13 mutants were localized predominantly to melanosomes (positive for pigment granules, TYRP1 and TYR), compared to tubular endosomes localized STX13^WT^, and absent from lysosomes (Fig. 3B,C,E; quantified in Fig. 3F, *r*=0.48±0.02 in STX13^WT^, 0.69± 0.02 in STX13^Δ14-129^ and 0.71±0.02 in STX13^Δ129^; mean±s.e.m.). Furthermore, a cohort of STX13 mutants were localized to EEA1-positive early endosomes similar to STX13^WT^ (arrows) and this co-distribution was reduced in STX13 mutants (Fig. 3D; quantified in Fig. 3G, *r*=0.45±0.03 in STX13^WT^, 0.37±0.02 in STX13^Δ14–129^ and 0.31±0.02 in STX13^Δ129^; mean±s.e.m.), indicating that these mutants are mislocalized to the melanosomes. Thus, these studies indicate that regulatory domain of STX13 is required for efficient recycling from melanosomes to endosomes.

### STX13 recycling from melanosomes requires an indirect role of AP-3 complex

**Fig. 4.**
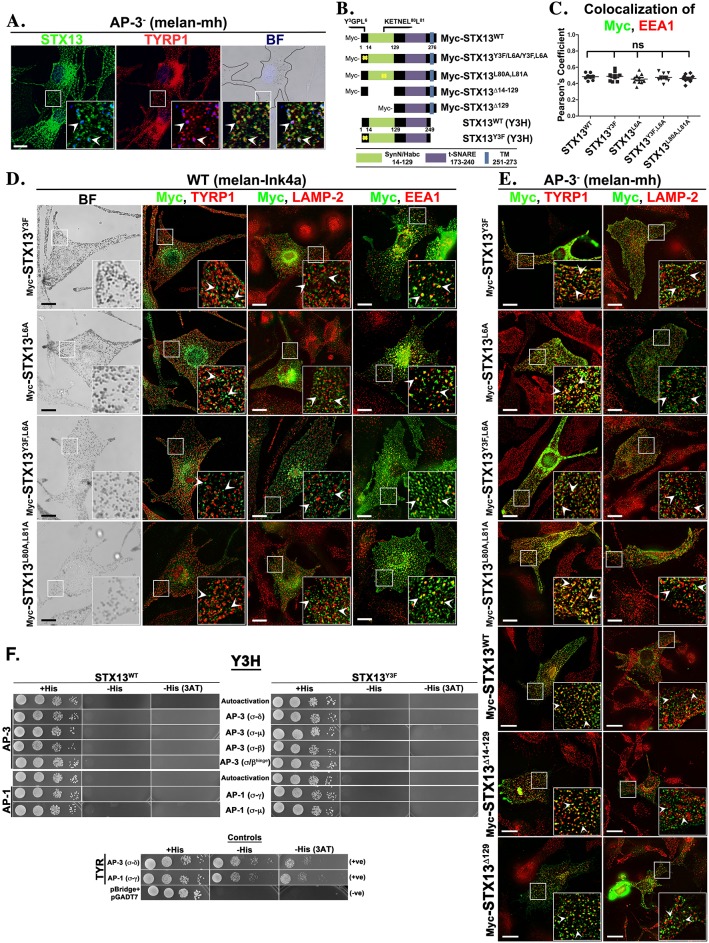
**AP-3 indirectly controls SNARE recycling from melanosomes.** (A) Bright-field (BF) and immunofluorescence microscopy images of AP-3^−^ (melan-mh) melanocytes. Arrowheads indicate the colocalization of STX13 with TYRP1 or bright-field melanosomes (pseudocolored blue). (B) Schematic representation of deletion or point mutations in the regulatory domain of STX13. (C–E) Bright-field and immunofluorescence microscopy images of wild-type (melan-Ink4a) (C,D) or AP-3^−^ (melan-mh) melanocytes (E) expressing different Myc–STX13 plasmids. Arrowheads point to the Myc–STX13 localization. Nuclei are stained with Hoechst 33258. The insets are a magnified view of the white boxed areas. Scale bars: 10 μm. The graph in C represents the degree of colocalization between STX13^WT^ or mutants and EEA1 (*n*=8–10 cells). ns, not significant. (F) Y3H interaction between STX13 or TYR (C-terminus tail) and AP-3 or AP-1 hemicomplexes. The yeast strain Y2HGold was transformed with the respective bait and prey plasmids as shown in the figure (also see supplementary material Table S1), and the transformants were selected on +His plates. For example, the yeast cells were transformed with bait plasmid encoding STX13 (WT or Y3F mutant) with either the σ3 or σ1 subunits, and prey plasmid encoding δ, μ3, β3, β^hinge^ of AP-3 or γ or μ1 subunits of AP-1. Note that the TYR (C-terminus tail) acts as a positive control and the empty vectors as negative control in the assay. The transformants were selected on +His, −His and −His (2 mM 3AT) plates for the reporter activity.

Previous studies have shown that melanocytes deficient for AP-3 subunit β3A (melan-pe) mislocalize the endogenous STX13 to melanosomes and have hypothesized that AP-3 regulates STX13 recycling from melanosomes (Setty et al., 2007). Here, we verified the mislocalization of STX13 to melanosomes in another melanocyte cell line deficient for AP-3 δ subunit (melan-mh) (Fig. 4A), indicating a role for AP-3 in SNARE recycling. However, the hypopigmentation observed in these cells is due to defective TYR transport (Theos et al., 2005), but not TYRP1, to melanosomes (Setty et al., 2007). We tested whether AP-3 requires any conserved motifs such as the Yxxφ or dileucines in STX13^WT^ for its recycling from melanosomes. We hypothesized that mutation or deletion of such motifs would mislocalize the SNARE to melanosomes in wild-type melanocytes. Bioinformatic motifs analysis identified two putative adaptor protein-binding motifs, Y^3^GPL^6^ (Yxxφ) and KETNEL^80^L^81^ (dileucine), in the STX13 sequence, that could potentially be recognized by AP-3 (Fig. 4B). Single or combined mutations in the Y^3^GPL^6^ or KETNEL^80^L^81^ motifs of Myc–STX13 had no impact on their localization to EEA1-positive endosomes or pigmentation and the mutants did not associate predominantly with either the melanosomal protein TYRP1 or lysosomal protein LAMP-2 (Fig. 4D, a quantification of the colocalization efficiency between Myc–STX13 and EEA1 is shown in Fig. 4C; *r=*0.48±0.01 in STX13^WT^, 0.49±0.02 in STX13^Y3F^, 0.46±0.02 in STX13^L6A^, 0.47±0.02 in STX13^Y3F,L6A^ and 0.46±0.01 in STX13^L80A,L81A^; ns, not significant; mean±s.e.m.). Thus, these mutants function in a similar manner to Myc–STX13^WT^ (Figs 2 and 3). Moreover, STX13 mutants (including Habc deleted) were mislocalized to TYRP1-positive melanosomes, but absent in lysosomes in the AP-3^−^ (melan-mh) melanocytes (Fig. 4E). Additionally, hypopigmentation of AP-3^−^ cells was not restored with the expression of active STX13 mutants (data not shown), suggesting that the sorting of TYR by AP-3 at the early endosomal domains is necessary for pigmentation.

Furthermore, we tested whether the N-terminus of STX13 directly interacts with AP-3 subunits (δ, β3 and μ3) (supplementary material Table S1). Studies have shown that C-terminal tail of TYR interacts with δ–σ3 of AP-3 or γ–σ1 of AP-1 hemicomplexes in a yeast tri-hybrid (Y3H) assay (Fig. 4F, for protein expression see supplementary material Fig. S3C, Table S1) (Theos et al., 2005). Similarly, we tested the interaction between STX13 and AP-3 or AP-1 hemicomplexes in an Y3H assay. Unexpectedly, the STX13 mutants (STX13^Δ129^, STX13^Δ14–129^ and STX13^Y3F,Δ14–129^) showed autoactivation on −His reporter assay plates with or without 2 mM 3AT (3-amino-1,2,4-triazole, a competitive inhibitor of the *HIS3* gene product) (data not shown for Y3H; mutants showed similar autoactivation in the Y2H assay, see supplementary material Fig. S3A,B). However, STX13^WT^ and STX13^Y3F^ did not display any autoactivation in the Y3H assay (Fig. 4F, for protein expression see supplementary material Fig. S3C). In addition, both these STX13 mutants showed no direct interaction with different combinations of AP-3 hemicomplexes (δ–σ3, μ3–σ3, β3A–σ3 or β3A^hinge^–σ3), as well as with AP-1 hemicomplexes (γ–σ1 or μ1–σ1) (Fig. 4F, supplementary material Table S1). Moreover, protein expression of these hemicomplexes and STX13 was not affected in yeast (supplementary material Fig. S3C), suggesting a possible indirect interaction between STX13 and AP-3, very likely in a SNAREpin complex.

### STX13–VAMP7 acts as fusion machinery for melanosome cargo transport

**Fig. 5.**
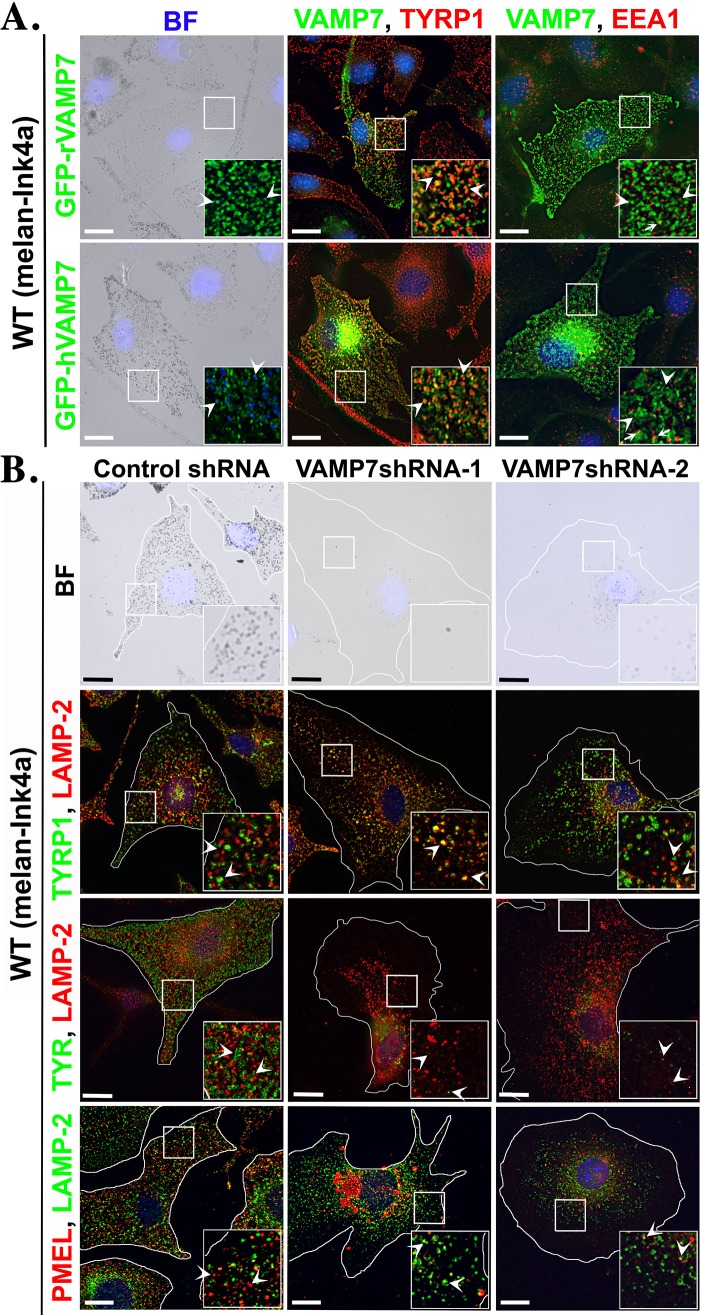
**GFP–VAMP7 localizes to melanosomes in wild-type melanocytes and its knockdown affects melanocyte pigmentation and cargo transport.** (A) Bright-field (BF) and immunofluorescence microscopy images of melanocytes expressing two different (rat or human) GFP-epitope tagged VAMP7 constructs. Melanosomes are pseudocolored blue. Arrowheads and arrows indicate the colocalization of GFP–VAMP7 with TYRP1 and EEA1, respectively. Note, the majority of GFP–VAMP7 localized to melanosomes and partly to the early endosomes in wild-type melanocytes. (B) Bright-field (BF) and immunofluorescence microscopy images of VAMP7-depeleted and control melanocytes. Arrowheads indicate the localization of TYRP1 or TYR or PMEL with respect to LAMP-2. Nuclei are stained with Hoechst 33258. The insets are a magnified view of the white boxed areas. Scale bars: 10 μm.

During fusion, SNAREs form SNAREpin complex by pairing Qa, Qb and Qc on one membrane (donor) with a distinct R-SNARE on another membrane (acceptor) (Fasshauer, 2003; Hong, 2005; Jahn and Scheller, 2006). We tested whether cargo transport mediated by an endosomal Qa-STX13 (in a complex with unknown Qb and Qc SNAREs), requires an R-SNARE on melanosomes. We focused our study on VAMP7, an R-SNARE reported to have multiple functions and that localizes to different subcellular compartments namely, endosomes, late endosomes, secretory vesicles and autophagosomes in non-melanocytic cells (Burgo et al., 2012; Fader et al., 2009; Kent et al., 2012; Pols et al., 2013). Additionally, VAMP7 has been shown to interact with STX13 in fibroblasts (Chung et al., 2013; Lu et al., 2013) and its knockdown in melanocytes affects the cargo transport to melanosomes (Bultema et al., 2014; Tamura et al., 2011; Yatsu et al., 2013). However, it is unclear from these studies whether VAMP7 functions as an R-SNARE on melanosomes for STX13-mediated transport pathways. Expression of GFP-tagged rat (r) or human (h) VAMP7 (VAMP7-TI) in wild-type melanocytes clearly showed both proteins predominantly localized to melanosomes and a minor cohort to EEA1-positive early endosomes (Fig. 5A), suggesting a role for VAMP7 on melanosome membranes.

**Fig. 6.**
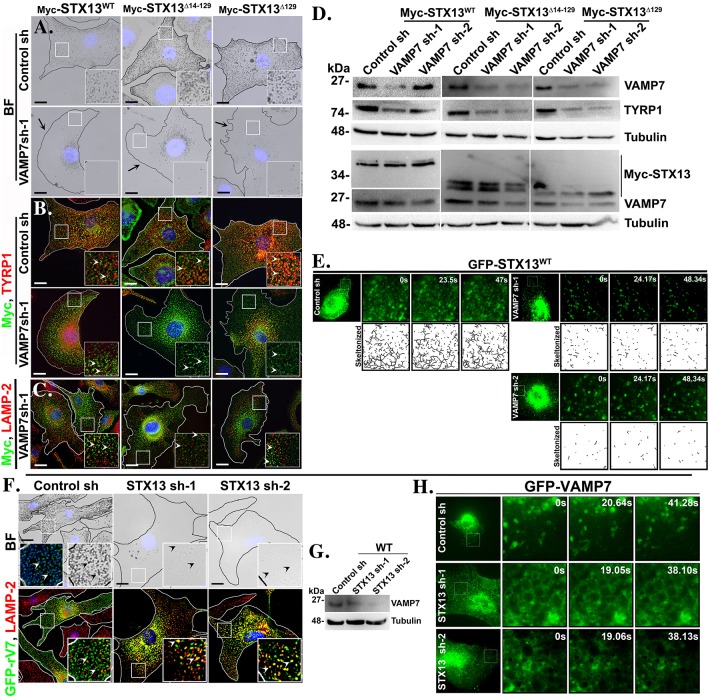
**STX13 and VAMP7 trafficking to melanosomes are interdependent on each other.** (A–D) Bright-field (BF) (A) and immunofluorescence microscopy (B,C) images, and immunoblotting (D) of melanocytes stably expressing Myc–STX13^WT^ or mutants after VAMP7 knockdown. Arrows indicate the loss of pigmentation in VAMP7-depleted cells (A) and arrowheads represent the Myc–STX13 localization (B,C). (E) Live imaging microscopy of GFP–STX13-expressing VAMP7-knockdown melanocytes. Insets represent GFP localization at different time points and their respective skeleton images. (F,H) Bright-field and immunofluorescence microscopy (F), and live imaging microscopy (H) images of GFP–VAMP7-expressing STX13-knockdown melanocytes. Arrowheads point to the localization of GFP–VAMP7 (F). Insets represent GFP localization at different time points (H). (G) Immunoblotting of STX13-depeleted melanocytes. Nuclei are stained with Hoechst 33258. The insets are a magnified view of the white boxed areas. Scale bars: 10 μm.

We tested the precise role of VAMP7 in melanocyte pigmentation by using VAMP7-specific shRNAs (VAMP7 sh-1 and VAMP7 sh-2). Similar to STX13-knockdown, VAMP7 depletion in melanocytes dramatically reduced the pigmentation (Tamura et al., 2011) and TYRP1 was targeted to the lysosomes. In addition, the expression levels of both TYR and PMEL were also reduced (Fig. 5B). These results indicate that VAMP7 regulates the cargo delivery to melanosomes in a similar manner to endosomal STX13. Furthermore, we tested whether the active STX13 mutants (Myc–STX13^Δ129^ or Myc–STX13^Δ14–129^) could rescue the pigmentation loss in VAMP7-knockdown cells. VAMP7 depletion in wild-type melanocytes stably expressing STX13 mutants or STX13^WT^ did not rescue the hypopigmentation (arrows) or TYRP1 expression (arrowheads) to melanosomes (Fig. 6A,B). Interestingly, Myc–STX13^Δ129^ and Myc–STX13^Δ14–129^ mutants appeared as endosomal puncta instead of melanosomal ring-like structures in VAMP7-depleted melanocytes (Fig. 6B). In contrast, the endosomal localization of Myc–STX13^WT^ was not substantially affected by VAMP7 depletion (Fig. 6B). Moreover, STX13^WT^ or its mutants were not targeted to the lysosomes, consistent with the unaffected protein levels in the VAMP7-knockdown cells (Fig. 6C,D), suggesting a crosstalk between STX13 and VAMP7 in regulating their steady-state localization. Overall, these results indicate that VAMP7 is required for STX13-mediated cargo transport as well as its trafficking to melanosomes.

To understand the cross-regulation between these two SNAREs, we further studied the localization of VAMP7 in STX13-depleted melanocytes. Surprisingly, GFP–rVAMP7 was targeted and degraded in lysosomes upon STX13 knockdown, whereas in control cells VAMP7 was localized to the melanosomes (Fig. 6F,G). Consistent with this, live imaging microscopy showed that GFP–VAMP7 was localized to an enlarged vacuolar structures (positive for LAMP-2, data not shown) in STX13-knockdown cells compared to a small ring-like melanosome structures in control melanocytes (Fig. 6H; supplementary material Movies 3–5). These results indicate that STX13 is required for localization of VAMP7 to melanosomes. In contrast, the localization of GFP–STX13^WT^ to endosomal tubular domains was unaffected by live-cell imaging microscopy, but these tubular structures were shorter in length in VAMP7-knockdown cells compared to the control cells (Fig. 6E; supplementary material Movies 6–8, see skeletonized form of GFP-STX13 localization), consistent with immunofluorescence microscopy studies (Fig. 6B). Furthermore, we tested the direct interaction between STX13 and VAMP7 in an Y2H assay (supplementary material Table S1). VAMP7 contains an N-terminal longin domain (amino acids 1–120) before the SNARE domain (supplementary material Fig. S3A) (Kent et al., 2012). We cloned the different domains and mutants of VAMP7 and STX13 (without transmembrane domain) into an Y2H vector and examined their autoactivation on Y2H reporter assay plates (supplementary material Fig. S3B, Table S1). Surprisingly, STX13^WT^ and its mutants, but not VAMP7 showed autoactivation on −His reporter assay plates (supplementary material Fig. S3B). However, the autoactivation of STX13^WT^ and STX13^Y3F^ was completely abolished on −His (3AT) plates compared to other mutants STX13^Δ129^, STX13^Δ14–129^ and STX13^Y3F,Δ14–129^ and hence these STX13 deletion mutants did not qualify for the Y2H assay (supplementary material Fig. S3B, includes protein expression). Interestingly, STX13 WT or Y3F mutant showed an interaction with wild-type VAMP7 (VAMP7^WT^) and with the SNARE (VAMP7^Δ120^) and longin domain (VAMP7^1–120^) of VAMP7 on −His but not on −His (3AT) reporter assay plates (supplementary material Fig. S3D, includes protein expression), indicating a false-positive interaction between these two SNAREs. Moreover, STX13–VAMP7 interaction on −His plates was equivalent to that of the STX13 autoactivation (supplementary material Fig. S3B). As expected, STX13 (WT or Y3F mutant) showed an interaction with pallidin (subunit of BLOC-1) on −His (3AT) plates, a positive control used in the assay (supplementary material Fig. S3B, Table S1). Furthermore, *in vivo* interaction between STX13 and VAMP7 using melanocyte cell lysates was negative (data not shown), meaning that it is plausible a very transient interaction between these two SNAREs might occur in SNAREpin complex. Taken together, these results suggest that both VAMP7 and STX13 regulate the localization of each other in addition to melanosomal cargo transport.

## DISCUSSION

Organelle maturation or biogenesis is crucially dependent on cargo transport mediated by specific SNARE proteins either in the form of vesicular or tubular fusion events. Melanosomes acquire cargo proteins for their biogenesis in two different transport steps from recycling endosomes (Marks et al., 2013; Sitaram and Marks, 2012). Although, several SNARE proteins have previously been implicated in melanosome maturation, our study for the first time identifies the function and regulation of two SNAREs, STX13 and VAMP7, in the biogenesis of this LRO.

Our results show that endosomal STX13 and melanosomal VAMP7 acts as key molecules in melanosomal transport, pigmentation and biogenesis. Data in this study also suggest that both SNAREs are essential for transport of endosomal tubular domains containing either TYRP1 or TYR cargo to melanosomes. In addition, their expression is also required for the biogenesis of PMEL-positive premature melanosomes. Previous studies in non-melanocytes have shown that STX13 is required for recycling of transferrin receptor and other cargo to cell surface (Prekeris et al., 1998), homotypic endosome fusion (McBride et al., 1999), phagophore maturation (Lu et al., 2013) and axon regeneration during nerve injury (Cho et al., 2014). By contrast, VAMP7 is involved in the fusion of late endosomes with lysosomes (Chaineau et al., 2009), delivery of GLUT1 to plasma membrane along with retromer (Hesketh et al., 2014), membrane repair (Rao et al., 2004), neurite outgrowth (Martinez-Arca et al., 2000) and fusion of autophagosomes with lysosomes (Fader et al., 2009). However, our study indicates that both STX13 and VAMP7 regulate the cargo transport from recycling endosomes to LROs, such as melanosomes, in melanocytes. Several observations support this conclusion that both SNAREs equally control the steps in melanosome formation by mutually regulating their localization: (1) we found that knockdown of either of these SNAREs resulted in loss of pigment granules, (2) SNARE depletion also caused the targeting of melanosome cargoes to lysosomes, (3) GFP–VAMP7 and a cohort of Myc– or GFP–STX13 proteins were localized to melanosomes, (4) STX13 knockdown led to the presence of enlarged VAMP7-positive vacuolar endosomes observed in live imaging analysis, and (5) VAMP7 knockdown affected the length of STX13-positive tubular intermediates. Finally, VAMP7 was targeted to the lysosomes for degradation in the absence of STX13, and STX13 was retained in the endosomes in the absence of VAMP7. Thus, this evidence supports a role for these SNAREs in the membrane trafficking steps to melanosomes.

As reported previously, and as also shown by our results, STX13 interacts very strongly with BLOC-1 (pallidin subunit) complex in a Y2H assay (supplementary material Fig. S3B) (Ghiani et al., 2010; Huang et al., 1999; Moriyama and Bonifacino, 2002). In addition, our previous studies have suggested that BLOC-1 recruits STX13 onto the endosomal domains that contain TYRP1 cargo. This is consistent with the accumulation of STX13 in vacuolar endosomes in BLOC-1-deficient melanocytes (Delevoye et al., 2009; Setty et al., 2007), indicating that BLOC-1 acts upstream of STX13. In contrast, our results also showed that AP-3 controls STX13 trafficking through a non-canonical binding motif, possibly in a SNARE complex with VAMP7. This is consistent with the known interaction between AP-3 (δ subunit) and VAMP7 (longin domain) in a cis-SNARE conformation (Kent et al., 2012), suggesting that AP-3 indirectly regulates STX13 recycling from melanosomes. Mutations in BLOC-1 or AP-3 complex subunits disrupt the transport of TYRP1 or TYR, respectively, to the melanosomes (Setty et al., 2007; Theos et al., 2005). However, to our surprise, STX13 depletion affected the melanosomal trafficking of both the cargoes (including PMEL, see below), suggesting that the SNARE functions in both BLOC-1- and AP-3-dependent transport steps to melanosomes. We hypothesized that STX13 regulates these transport pathways, possibly by interacting with different cognate SNAREs. This is consistent with our immunoelectron microscopy and live-cell imaging data, which shows the co-distribution of STX13 with TYR on endosomal tubular domains. Furthermore, the targeting of PMEL to lysosomes was presumably an indirect effect of STX13 knockdown, which causes the accumulation of autophagosomes in fibroblast cells (Lu et al., 2013).

Our results support a model wherein endosomal STX13 (with its unknown Qb and Qc SNAREs) form a SNAREpin complex with melanosomal VAMP7 to mediate the cargo transport to maturing melanosomes. This model is consistent with the notion that loss of expression of either SNARE would result in degradation of structural melanosomal proteins in the lysosome, which will further affect the formation of pre- or maturing melanosomes. In addition, this model is supported by recent findings in fibroblasts and neuronal cells: (1) STX13 interacts biochemically and forms a SNARE complex with VAMP7 for the transport of NCC (the Na^+^Cl^−^ co-transporter) to lysosomes (Chung et al., 2013); and (2) the loss of STX13 or VAMP7 accumulate autophagosomes (Cho et al., 2014; Fader et al., 2012; Lu et al., 2013). Studies have also shown that VAMP7 (R) forms a complex with STX3 (Qa) in melanocytes (Yatsu et al., 2013) and with STX7 (Qa) in rat liver lysates (Kent et al., 2012; Pryor et al., 2004). This suggests that VAMP7 controls multiple transport steps depending on its localization and pairing SNARE partners. We are currently developing tools to test the mechanism of SNARE pairing of STX13 and VAMP7 in different cargo transport steps to melanosomes. We speculate that STX13 (Qa) very likely regulates the melanosome trafficking steps by pairing with different Qb and Qc SNAREs on transport intermediates containing distinct melanosome cargo. Nevertheless, the precise role of the predicted cognate Qb and Qc SNARE partners of STX13 such as SNAP25 (Qbc) (Chung et al., 2013; Ghiani et al., 2010; Yatsu et al., 2013), Vti1a (Qb) (Lu et al., 2013), Vti1b (Qb) or STX8 (Qc) (Lu et al., 2009) in melanosome pigmentation needs to be validated. Overall, our study shows that STX13 and VAMP7 coordinately regulate melanosome maturation by controlling endosomal cargo transport to the melanosomes.

## MATERIALS AND METHODS

### Chemicals and tissue culture reagents

3-Amino-1,2,4-triazole (3AT), copper (II) sulfate pentahydrate (copper), 3,4-Dihydroxy-L-phenylalanine (L-DOPA), Bafilomycin A1, polybrene (hexadimetrine bromide), Hoechst 33258 and protease inhibitor cocktail tablets were from Sigma-Aldrich. Hygromycin B, Lipofectamine 2000 and all other tissue culture reagents were from Life Technologies (Invitrogen).

### Antibodies

Polyclonal antisera against the following proteins were used: Rab5 (Cell Signaling Technology); TYRP1 (H-90), c-Myc (A-14 for immunoblotting) and EEA1 (goat) (Santa Cruz Biotechnology). Other antisera to VAMP7 (a gift from Andrew Peden, University of Sheffield, Sheffield, UK); STX13 (Prekeris et al., 1998); and TYR (Theos et al., 2005) are as described previously. Monoclonal antisera against the following proteins were used: PMEL (HMB45, Abcam); TYRP1 (TA99, ATCC); adaptin γ (BD Biosciences, AP-1); adaptin δ (SA4, AP-3 used for immunofluorescence microscopy), LAMP-1 (1D4B), LAMP-2 (GL2A7) and c-Myc (9E10) (Developmental Studies Hybridoma Bank); adaptin σ3 (Santa Cruz Biotechnology); γ-tubulin (Sigma-Aldrich). Secondary antibodies were either from Molecular Probes (Life Technologies) or Jackson Immunoresearch.

### DNA, yeast two- or tri-hybrid and shRNA constructs

#### STX13 expression constructs

Myc–STX13^WT^ – human full-length STX13 – was PCR amplified with a N-terminal Myc epitope sequence from an I.M.A.G.E. clone (3851266, obtained from ATCC, represented as STX12) and subcloned into the BamHI and XhoI sites of both pcDNA3.1(+) (Invitrogen) and pBMN-IRES-Hygro (a retroviral vector, gift from Richard Scheller, Genentech). Similarly, N-terminal deletion mutants, Myc–STX13^Δ14–129^ (deletion of amino acids 14–129) and Myc–STX13^Δ129^ (deletion of amino acids 1–129) were cloned in the above vectors. Mutagenesis of N-terminal amino acids Y3F (Myc–STX13^Y3F^), L6A (Myc–STX13^L6A^), Y3F and L6A (Myc–STX13^Y3F,L6A^), L80A and L81A (Myc–STX13^L80A,L81A^) in Myc–STX13^WT^ (pBMN-IRES-Hygro) was carried out using the QuikChange multi site-directed mutagenesis kit (Agilent Technologies). GFP–STX13^WT^ and GFP–STX13^Δ129^ – full-length and N-terminal deletion containing amino acids 1–129 of human STX13 – were PCR amplified, digested with BamHI and XhoI enzymes and subcloned into the BglII and SalI sites of pEGFP-C1 (Clontech). RFP–STX13, mRFP and STX13 were PCR amplified separately, digested with BamHI and EcoRI, and EcoRI and XhoI enzymes, respectively, and subcloned into pcDNA3.1(+) at BamHI–XhoI sites.

#### Yeast two- and tri-hybrid constructs

All vectors used for studying the interaction of STX13 or its mutants with AP-3 or AP-1 hemicomplexes using Y3H, or with VAMP7 using Y2H are described in supplementary material Table S1.

#### Other constructs

GFP–rVAMP7 has been described previously (Puri et al., 2003). GFP–hVAMP7 (Martinez-Arca et al., 2000) and GFP–TYR (Halaban et al., 2000) were obtained from Addgene.

#### STX13 and VAMP7 shRNA vectors

Oligodeoxyribonucleotide duplexes containing the target sequences were cloned into the BamH1 and HindIII sites of pRS shRNA vector (OriGene Technologies). The following sequences were selected as targets: STX13 shRNA-1, 5′-AAATCAGCTCGCCAAGGAAAC-3′ (from nucleotide 210); STX13 shRNA-2, 5′-AAAGGTATCTGAGAAGGAAAA-3′ (from nucleotide 366), VAMP7 shRNA-1, 5′-TAAGAGCCTAGACAAAGTGAT-3′ (from nucleotide 360); and VAMP7 shRNA-2, 5′-TCGAGCCATGTGTATGAAGAA-3′ (from nucleotide 537). Empty pRS shRNA plasmid was used as a control in all shRNA knockdown experiments.

#### Primers and sequencing

Mouse-specific TYRP1 (5′-CCCCTAGCCTATATCTCCCTTTT and 5′-GCCCTGACAAAGTGGCTCT), TYR (5′-ATCAGCTCAGTCTATGTCATCCC and 5′-TGCCAAGGCAGAAACCCTGGT), STX13 (5′-ATGTCCTACGGTCCCTTA and 5′-TCATTTAGAAGCAACCCA) and Myc–STX13 (5′-CTGAAGAAGACTTGGAATTC and 5′-TCACTTCGTTTTATAAACTAG) primers were used for the semiquantitative PCR. All plasmid inserts were verified by DNA sequencing.

### Yeast two- or tri-hybrid assay

The yeast strain Y2HGold (Clontech) was maintained on YPD (yeast extract, peptone, dextrose) plates. Transformation of different bait and prey plasmids (supplementary material Table S1) in the Y2HGold was performed by a modified lithium acetate procedure as described in the Yeast Two-Hybrid System book (Golemis and Brent, 1997). The yeast transformants were selected on minimal medium plates supplemented with yeast synthetic drop-out amino acid mix (Y0750 from Sigma-Aldrich) lacking leucine and tryptophan (referred to here as +His or +Histidine medium). For the reporter assay, transformants were grown to an absorbance of 0.4–0.5 at 600 nm and then spotted on plates that were +His, −His (−His medium, Y2146 from Sigma-Aldrich) and −His containing 2 mM 3AT after serial diluting the cultures by tenfold in sterile water. Plates were grown at 30°C for 3–5 days and then imaged under white light in a Bio-Rad Molecular Imager. Note that the STX13 (WT and Y3F) constructs showed autoactivation on −His, but not on −His (3AT) reporter plates (supplementary material Fig. S3B). Thus, growth of yeast transformants on −His (3AT) reporter plates was considered as criteria for protein–protein interaction.

### Cell culture, transfection and retroviral transduction

Immortal melanocyte cell lines were used in this study – wild-type melan-Ink4a-Arf-1 (from C57BL/6J, *a/a*, *Ink4a-Arf^−/−^* mice*,* formerly called melan-Ink4a-1, referred to here as WT or melan-Ink4a) (Ha et al., 2007), and AP-3-deficient melan-mh1 [from C57BL/6J *Ap3d^mh^*^/*mh*^, referred to here as AP-3^−^ (melan-mh)] or melan-pe1 [from C57BL/6J *Ap3b1^pe/pe^*, referred to here as AP-3^−^ (melan-pe)] (Theos et al., 2005). Cells were maintained as described previously (Sviderskaya et al., 2002).

Plasmids were transfected into the melanocytes or PLAT-E cells (Cell Biolabs) by Lipofectamine 2000 (Invitrogen) according to the manufacturer's protocol. Melanocytes were also transduced with retroviruses (containing different pBMN-IRES-Hygro or pRSshRNA plasmids) isolated from PLAT-E cells (Morita et al., 2000). Post transduction, melanocytes were selected twice with hygromycin (200 μg/ml) or puromycin (2 μg/ml) on the 2nd and 4th day. In some experiments, shRNA knockdown cells were transfected with GFP–STX13 or GFP–VAMP7 using Lipofectamine 2000 reagent. Stable wild-type (melan-Ink4a) melanocytes expressing different constructs of Myc–STX13 were generated by the retroviral transduction method and used for immunoelectron microscopy and VAMP7 knockdown in Fig. 6A–D. STX13-knockdown cells were rescued by transfecting the cells with GFP–hSTX13^WT^.

### Transcript analysis by semiquantitative PCR

RNA was isolated from melanocytes by the Trizol method. Briefly, confluent cells in a 60-mm dish were treated with Trizol reagent (Sigma-Aldrich) and then RNA was extracted with chloroform at room temperature. The aqueous layer was precipitated with isopropanol followed by a wash with 70% ethanol. Finally, the RNA pellet was air dried, suspended in 0.01% DEPC-treated water (Sigma-Aldrich) and the concentration estimated using a NanoDrop 2000C spectrophotometer (Thermo Scientific). cDNA was prepared by using a cDNA synthesis kit (Fermantas). Transcript levels of a gene were analyzed by PCR (Bio-Rad S1000 Thermal Cycler) using gene specific primers and an equal amount of cDNA from each sample. GAPDH was used as a control in the PCR.

### Immunoblotting

Melanocyte cell lysates for immunoblotting were prepared using a protocol described previously (Setty et al., 2007). In all blotting experiments, γ-tubulin was used as a loading control. Immunoblots were developed either with Luminata Classico HRP substrate (Millipore) or Clarity Western ECL substrate (Bio-Rad) and the luminescence was captured using Image Lab 4.1 software in a Bio-Rad Molecular Imager ChemiDoc XRS+ imaging system equipped with Supercooled (−30°C) CCD camera (Bio-Rad). Protein band intensities were quantified, normalized and then plotted.

Yeast cell extracts were prepared using a modified protocol described previously (Baerends et al., 2000). Briefly, transformants were grown overnight in +His liquid medium, harvested (4,000 ***g***, 5 min, 4°C) and washed once with sterile water. The pellets were resuspended in 1 ml of 12.5% trichloroacetic acid (TCA), incubated for 30 min at −80°C and then pelleted at room temperature (16,800 ***g*** for 5 min). The pellets were washed twice with 1 ml of ice-cold acetone, air dried, suspended in 100 μl of 1% SDS, 0.1 M NaOH solution and then mixed with 100 μl of 2× SDS-PAGE sample buffer.

### Estimation of melanin pigments

The isolation and estimation of melanin pigments from mouse melanocytes was as described previously (Wasmeier et al., 2006). Briefly, cells were lysed in lysis buffer (50 mM Tris-HCl pH 7.4, 2 mM EDTA, 150 mM NaCl, 1× protease inhibitors) by sonication, pelleted, washed once with ethanol and diethyl ether mixture (1:1) and then air dried. Then the pellet was solubilized in a buffer (2 M NaOH, 20% DMSO) at 60°C for 15–30 min. Melanin pigments were estimated by measuring the absorbance at 492 nm and results were normalized to the protein concentration.

### *In vitro* tyrosinase activity

Tyrosinase activity in melanocytes was assayed using DOPA cytochemistry as described previously (Setty et al., 2008). The assay was performed in PBS (untreated) or PBS containing 0.1% L-DOPA with or without 20 μM copper sulphate.

### Protease inhibitor assay

Cells on a Matrigel-coated coverslip were treated with or without 50 nM bafilomycin A1 for 4 h at 37°C. Cells were washed with plain medium, and then with 1× PBS. Finally, cells were fixed with formaldehyde, stained with the respective antibodies and analyzed by immunofluorescence microscopy.

### Immunoprecipitation of GFP–STX13

Wild-type melanocytes expressing GFP or GFP–STX13^WT^ or GFP–STX13^Δ129^ were subjected to immunoprecipitation of GFP using the protocol described in the GFP-Trap kit (Chromotek). Briefly, cells were lysed in 1× RIPA buffer (10 mM Tris-HCl pH 7.5, 150 mM NaCl, 5 mM EDTA, 0.1% SDS, 1% Triton X-100, 1% deoxycholate and protease inhibitor cocktail) on ice for 30 min and then centrifuged at 20,000 ***g*** for 10 min at 4°C. The cell lysates were incubated with equilibrated GFP-Trap_A beads for 5 h under constant mixing at 4°C. The beads were then washed twice with wash buffer (10 mM Tris-Cl pH 7.5, 150 mM NaCl, 0.5 mM EDTA), suspended in 2× SDS-sample buffer and then subjected to immunoblotting.

### Electron microscopy

Cells were fixed with a mixture of 2% (w/v) paraformaldehyde and 0.2% (w/v) glutaraldehyde in 0.1 M PHEM buffer (120 mM PIPES, 50 mM HEPES, 4 mM MgCl_2_, 20 mM EGTA pH 6.9), and processed for immunoelectron microscopy as described previously (Setty et al., 2007). Ultrathin cryosections were prepared using UC7 ultracryomicrotome (Leica, Vienna, Austria) (Raposo et al., 1997), double immunogold labeled with protein A conjugated to 10- or 15-nm gold particles (PAG10, PAG15 from Cell Microscopy Center, AZU, Utrecht, The Netherlands) and analyzed under a Tecnai Spirit electron microscope (FEI, Eindoven, The Netherlands) equipped with a 4k CCD camera (Quemesa, Olympus).

### Immunofluorescence microscopy and image analysis

Cells were stained with primary antibodies followed by the respective secondary antibodies as described previously (Setty et al., 2007). Cells were imaged by bright-field and immunofluorescence microscopy using a 60× (oil) U Plan super apochromat objective on an Olympus IX81 motorized inverted fluorescence microscope equipped with a CoolSNAP HQ2 (Photometrics) CCD camera. Images were deconvolved and analyzed with the cellSens Dimension package with the 5D module. Cellular pigmentation was quantified visually (normal or hypopigmentation) by counting ∼100 cells in each experiment from bright-field images that were taken randomly from the sample at identical camera settings. Average pigmentation was calculated and then plotted. The colocalization coefficient between two colors was measured by selecting the entire cell, excluding the perinuclear area, and estimating the Pearson's correlation coefficient (*r*) value using cellSens Dimension and then plotted. Note that the maximum intensity projection of undeconvolved *z*-stack images were used for estimating the *r* values. The analyzed images were assembled using Adobe Photoshop.

### Live cell imaging of GFP–STX13 or GFP–VAMP7 in control and knockdown cells or GFP–TYR and RFP–STX13 in wild-type melanocytes

Wild-type (melan-Ink4a) melanocytes were plated on 2-cm glass-bottomed dishes (MatTek Corp.) and then transfected with either the GFP–STX13^WT^ or GFP–STX13^Δ129^ construct. After 48 h, cells were visualized with an Olympus IX81 fluorescence microscope equipped with an environmental chamber maintained at 37°C with 5% CO_2_. Video microscopy of GFP and time-lapse microscopy of both GFP and bright-field melanosomes were performed by capturing image streams over 3–5 min using a CoolSNAP HQ2 (Photometrics) CCD camera. Images were analyzed with cellSens Dimension and processed into binary format and then skeletonized using ImageJ (NIH) software. Similarly, GFP–STX13^WT^ or GFP–rVAMP7 was transfected in control and VAMP7- or STX13-knockdown cells, respectively, and viewed using live imaging microscopy. Furthermore, wild-type melanocytes co-transfected with GFP–TYR and RFP–STX13 were analyzed by time-lapse live imaging microscopy.

### Statistical analysis

Statistical significance was determined by an unpaired Student's *t*-test and variance analysis using GraphPad software. ns, not significant; *P<0.05; **P<0.01 and ***P<0.001.

## Supplementary Material

Supplementary Material
