# Supplementary material for: STX13 regulates cargo delivery from recycling endosomes during melanosome biogenesis
Source: J Cell Sci. 2015 Sep 1;128(17):3263–76. doi: 10.1242/jcs.171165 (PMC4582192; doi:10.1242/jcs.171165)
Supplement: Supplementary Material [file supp_128_17_3263__index.html]

STX13 regulates cargo delivery from recycling endosomes during melanosome biogenesis — Supplementary Material 

# STX13 regulates cargo delivery from recycling endosomes during melanosome biogenesis

## JCS171165 Supplementary Material

- Supplementary Material
