# Supplementary material for: STX13 regulates cargo delivery from recycling endosomes during melanosome biogenesis
Source: J Cell Sci. 2015 Sep 1;128(17):3263–76. doi: 10.1242/jcs.171165 (PMC4582192; doi:10.1242/jcs.171165)
Supplement: Supplementary Material [file supp_jcs.171165_JCS171165supp.pdf]

Supplementary Information:

Table S1. Yeast two- and tri- hybrid constructs used in this study.

	Bait plasmids	Prey plasmids
**Y3H**	pBridge - AP-3(σ3) (-ve control)<br>pBridge - AP-3(σ1) (-ve control)<br>pBridge – TYR - AP-3(σ3) (+ve control)<br>pBridge - TYR - AP-1(σ1) (+ve control)<br>pBridge - $STX13^{WT}$ - AP-3(σ3)<br>pBridge - $STX13^{Y3F}$ - AP-3(σ3)<br>pBridge - $STX13^{WT}$ - AP-1(σ1)<br>pBridge - $STX13^{Y3F}$ - AP-1(σ1)	pGAD424 (empty vector control)<br>pGAD424–Pallidin ($pa^{110-172}$, BLOC-1)<br>pGADT7 (empty vector control)<br>pGADT7 – AP-3(δ)<br>pACT2 – AP-3(μ3)<br>pGADT7 – AP-3(β3A)<br>pGADT7 – AP-3(β3A-hinge)<br>pGADT7 – AP-1(γ)<br>pACT2 – AP-1(μ1)<br>pGADT7 – $VAMP7^{WT}$<br>pGADT7 – $VAMP7^{\Delta120}$<br>pGADT7 – $VAMP7^{1-120}$
**Y2H**	pGBKT7 (empty vector control)<br>pGBKT7 – $STX13^{WT}$<br>pGBKT7 – $STX13^{Y3F}$<br>pGBKT7 – $STX13^{\Delta129}$<br>pGBKT7 – $STX13^{\Delta14-129}$<br>pGBKT7 – $STX13^{Y3F,\Delta14-129}$	

<u>Plasmid construction:</u> Basic Y2H vectors (pGBKT7, pGAD424, pGADT7, pACT2) and Y3H vector (pBridge) were from the Matchmaker GAL4 based Y2H or Y3H system, obtained from Clontech Laboratories. All STX13 (human) constructs without transmembrane domain (Fig. S3A; $STX13^{WT}$: amino acids 1-249; $STX13^{Y3F}$: amino acids 1-249 with Y3F mutation; $STX13^{\Delta129}$: amino acids 130-249; $STX13^{\Delta14-129}$: amino acids 1-249 with a deletion from amino acids 14 to 129; $STX13^{Y3F,\Delta14-129}$: amino acids 1-249 with a deletion from amino acids 14 to 129 and Y3F mutation; $STX13^{1-129}$: amino acids 1-129; and $STX13^{1-129(Y3F)}$: amino acids 1-129 with Y3F mutation) were PCR amplified and cloned into EcoRI and SalI sites of pGBKT7 or MCS I of pBridge vector. All VAMP7 (human) constructs without transmembrane domain (Fig. S3A; $VAMP7^{WT}$: amino acids 1-189; $VAMP7^{\Delta120}$: amino acids 121-189; and $VAMP7^{1-120}$: amino acids 1-120) were PCR amplified and cloned into EcoRI and BamHI sites of pGADT7 vector. Beta subunit (full length, β3A, 1-1094 aa) and beta-hinge region (amino acids 576-902) of AP-3 were PCR amplified and cloned into SfiI and BamHI sites of pGADT7 vector. Subunits of AP-1 (γ or μ1 or σ1), AP-3 (δ or μ3 or σ3), BLOC-1 (human Pallidin, pa, amino acids 110-172) and TYR (mouse Tyrosinase C-terminus) in pGADT7 or pACT2 or pGAD424 or pBridge were described previously (Chaudhuri et al., 2007; Janvier et al.,

2003; Moriyama and Bonifacino, 2002; Sitaram et al., 2012; Theos et al., 2005). Note that TYR and sigma subunits of AP-3 or AP-1 were cloned in MCS I and II respectively in pBridge vector.

Y2H assay with negative controls: Empty vectors pGBKT7 (bait), a GAL4-DNA binding domain containing vector and pGADT7 (prey), a GAL4-activation domain containing vector were transformed into Y2HGold yeast strain. The obtained colonies were assayed for reporter activity and also tested for the expression of fusion proteins (Fig. S3B).

Y2H autoactivation assay: Y2HGold yeast strain was transformed with pGBKT7 vector containing wild-type or different mutants of STX13 and an empty pGADT7 vector. The obtained colonies were assayed for reporter activity and tested for the expression of fusion proteins (Fig. S3B). Similarly, Y2HGold yeast strain was transformed with an empty pGBKT7 vector and pGADT7 containing wild-type or different mutants of VAMP7 for testing the autoactivation of VAMP7 constructs (Fig. S3B, includes protein expression of VAMP7). Note that all STX13 constructs showed autoactivation on –His reporter plates (Fig. S3B). However, $STX13^{WT}$ and $STX13^{Y3F}$ constructs abolished their autoactivation on –His (3AT) plates compared to the other deletion mutants, indicating that $STX13^{\Delta 129}$, $STX13^{\Delta 14\text{-}129}$ and $STX13^{Y3F,\ \Delta 14\text{-}129}$ constructs are not suitable for Y2H or Y3H assay.

Y2H assay with positive control, STX13-BLOC-1 interaction: Y2HGold yeast strain was transformed with pGBKT7 vector containing wild-type or Y3F mutant of STX13 and pGAD424 containing pallidin (a BLOC-1 subunit, $pa^{110\text{-}172}$). The obtained colonies were assayed for reporter activity (Fig. S3B) (Moriyama and Bonifacino, 2002).

Y2H interaction between STX13 and VAMP7: Y2HGold yeast strain was transformed with either pGBKT7 vector containing wild-type or Y3F mutant of STX13 and pGADT7 vector containing wild-type or different mutants of VAMP7. The obtained colonies were assayed on –His and -His containing 3AT (2mM) reporter activity plates and also tested for the expression of fusion proteins (Fig. S3D).

Y3H assay with negative controls: Empty vectors pBridge (bait), a GAL4-DNA binding domain containing vector and pGADT7 (prey), a GAL4-activation domain containing vector were transformed into Y2HGold yeast strain. The obtained colonies were assayed for reporter activity (Fig. 4F).

Y3H autoactivation assay: Y2HGold yeast strain was transformed with pBridge vector containing either wild-type or Y3F mutant of STX13 at MCS I site and sigma3 or sigma1 at MCS II site and an empty pGADT7 vector. The obtained colonies were assayed for reporter activity (Fig. 4F) and tested for the expression of fusion proteins (Fig. S3C).

Y3H assay with positive controls, interaction between TYR and AP-3 or AP-1 hemicomplexes: Y2HGold yeast strain was transformed with pBridge vector containing TYR at MCS I site and sigma3 or sigma1 at MCS II site and pGADT7 vector containing AP-3 delta or AP-1 gamma subunits respectively. The obtained colonies were assayed for reporter activity (Fig. 4F) and tested for the expression of fusion proteins (Fig. S3C) (Sitaram et al., 2012).

Y3H interaction between STX13 and AP-3/AP-1 hemicomplexes: Y2HGold yeast strain was transformed with pBridge vector containing wild-type or Y3F mutant of STX13 at MCS I site and sigma3 or sigma1 at MCS II site and pGADT7 or pACT2 containing different subunits of AP-3 or AP-1 respectively. The obtained colonies were assayed for reporter activity (Fig. 4F) and tested for the expression of fusion proteins (Fig. S3C).

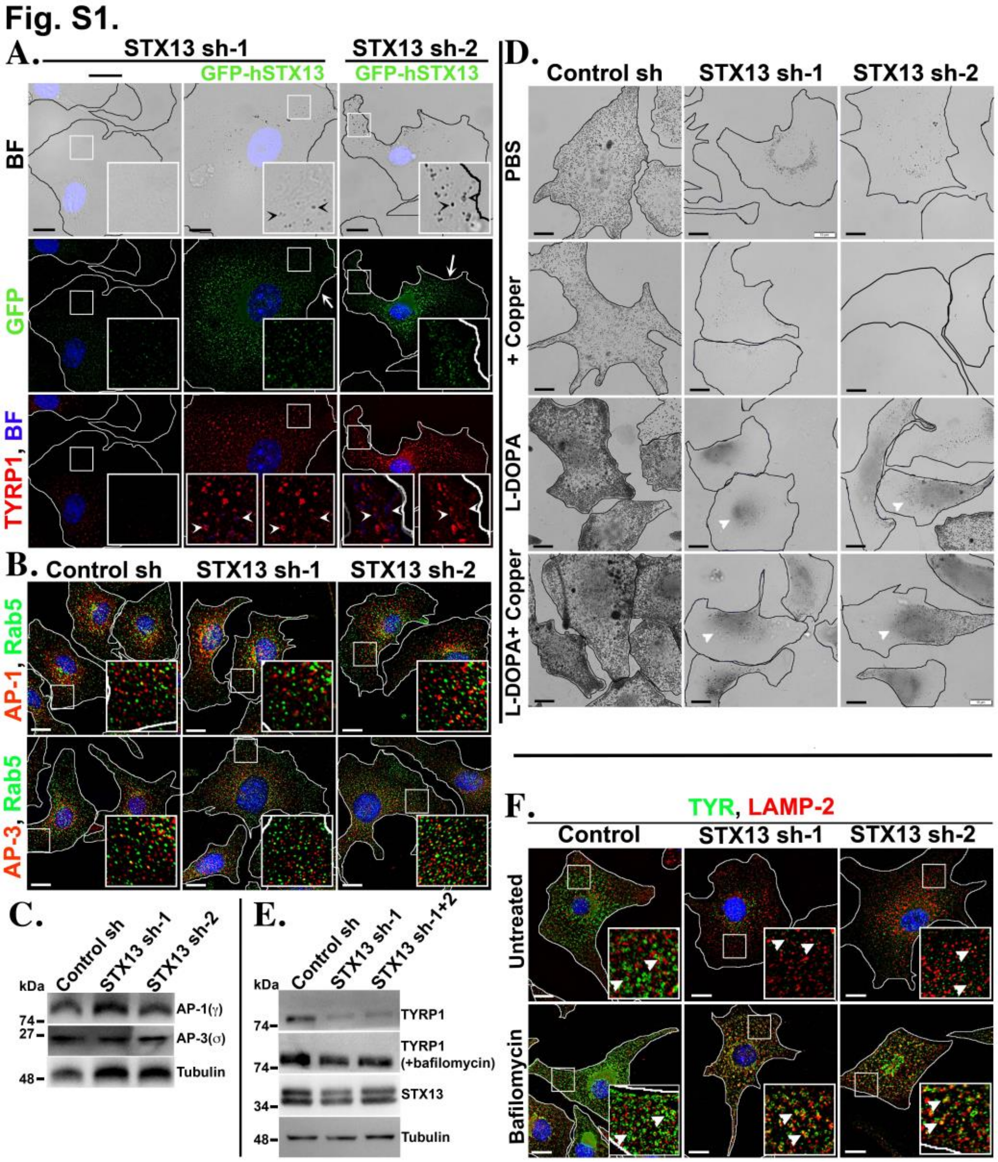

Fig. S1. (A) GFP-hSTX13 expression partially rescues the pigmentation and melanosome transport in STX13-knockdown mouse melanocytes. Bright-field microscopy and immunofluorescence microscopy images of untransfected or GFP-hSTX13 transfected STX13-depleted (shRNA-1 or shRNA-2) melanocytes. Arrows point to the increase in GFP-STX13 expression and arrowheads indicate the localization of TYRP1 with respect to bright-field melanosomes, pseudocolored to blue. **(B-C) STX13-knockdown unaffected the localization and expression of endosomal proteins AP-1, AP-3 and Rab5 in melanocytes.** Immunofluorescence microscopy image (B) and immunoblotting (C) of STX13-depleted melanocytes. **(D) STX13-knockdown affects**

the tyrosinase activity in melanocytes. Bright-field microscopy images of STX13-depleted melanocytes after addition of L-DOPA with or without copper (20 μM). Images were captured at identical camera setting. Arrowheads indicate a minor tyrosinase activity near the perinuclear region in L-DOPA treated cells (with or without copper). **(E-F) Bafilomycin treatment stabilized the TYRP1 expression and enhanced the staining of lysosomal targeted TYR in STX13-knockdown melanocytes.** Immunoblotting (E) and immunofluorescence microscopy images (F) of STX13-depleted melanocytes before and after the treatment of bafilomycin A1. Arrowheads indicate the localization of TYR with respect to LAMP-2. Nucleus was stained with Hoechst 33258. The insets are a magnified view of the white boxed areas. Scale bars, 10 μm.

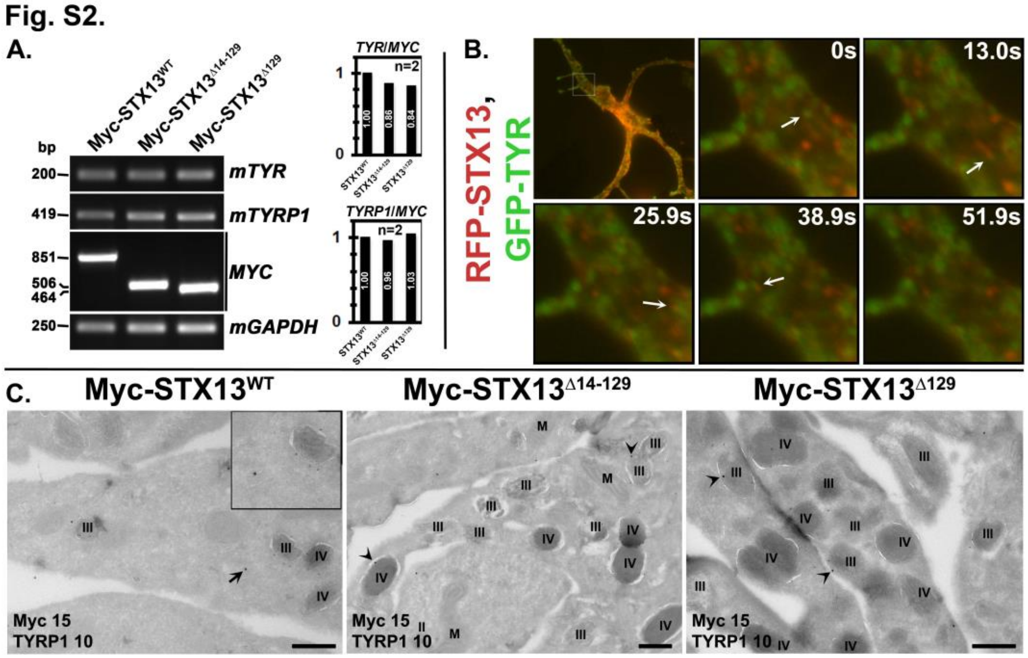

Fig. S2. (A) Overexpression of STX13 unaffected the transcript levels of melanosomal proteins in wild-type melanocytes. Transcript analysis of melanocytes expressing wild-type or mutant STX13 constructs using semiquantitative PCR as described in Materials and Methods. *MYC* expression represents the transcript levels of overexpressed Myc-STX13 and was measured using a primer specific to Myc-epitope and STX13. Graphs represent the fold changes in transcript levels of melanosomal proteins with Myc-STX13 over expression. Note that the DNA band intensities of TYRP1, TYR and Myc-STX13 were normalized with their respective GAPDH expression and plotted the ratio between TYRP1 or TYR with Myc-STX13 expression. **(B) A subset of melanosomal protein TYR localizes to STX13-positive structures.** Live cell imaging of GFP-TYR and RFP-STX13 in wild-type melanocytes. Arrows indicates the localization of TYR to STX13-postivie punctate or tubular compartments. **(C) Regulatory-domain-deficient STX13 mutants localizes to melanosomes.** Immunoelectron microscopy images of melanocytes stably expressing Myc-STX13^{WT} or mutants. Myc (PAG15) and TYRP1 (PAG10) represent the immunogold labeling of proteins with respective antibodies. Arrow and arrowheads represent the localization of STX13 to tubular endosomal domains and melanosomes (Stages III or IV) respectively. Inset, emphasize STX13- positive endosomal tubular structure closely associated to melanosome. M, mitochondria; II, III and IV, stages of melanosomes. Scale bars, 400 nm.

Fig. S3.

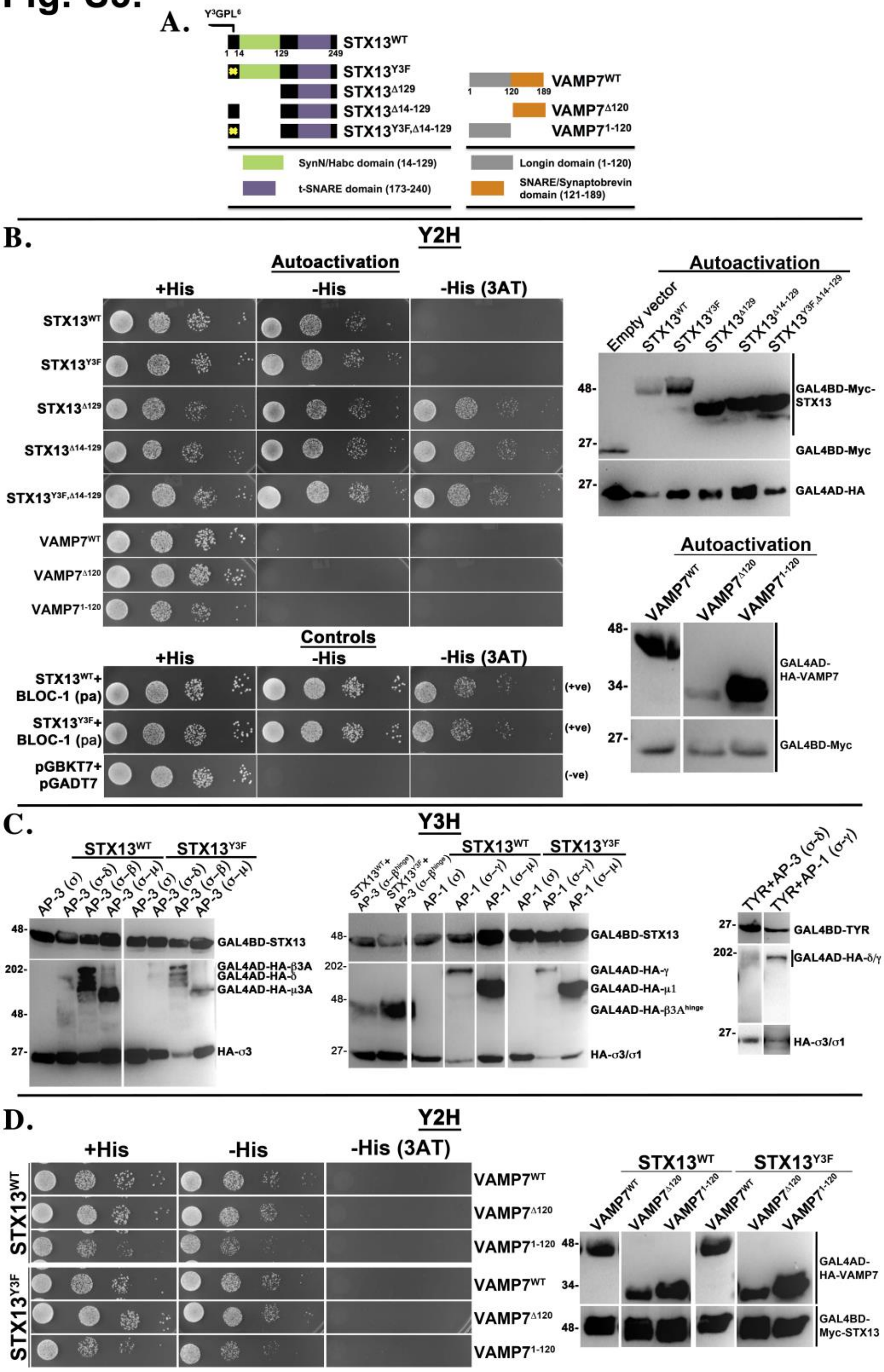

Fig. S3. (A) Conserved domains in STX13 and VAMP7 proteins. Schematic representation of point or deletion mutagenesis in STX13 and VAMP7 for Y2H or Y3H

assays. **(B) STX13 deletion mutants but not VAMP7 showed autoactivation in Y2H assay.** Transformed the yeast strain Y2HGold with STX13 bait and empty prey plasmids or empty bait and VAMP7 prey plasmids as shown in the figure (also see Table S1) and selected the transformants on +His plates. For positive control, transformed the yeast cells with STX13 (WT or Y3F mutant) bait plasmid and pallidin (a BLOC-1 subunit) prey plasmid, and for negative control, transformed the yeast cells with empty bait and prey plasmids separately. Selected the transformants on +His, –His and –His (2 mM 3AT) reporter activity plates. Note that the STX13 mutants Δ129, Δ14-129 and Δ14-129 with Y3F showed autoactivation on –His (3AT) plates in the assay. Yeast transformants expressing respective bait and prey plasmids as shown in the figure were lysed using a protocol described in Materials and Methods and then subjected to immunoblotting. Blots were probed with anti-Myc or anti-HA antibodies. **(C) Analysis of bait and prey protein expression in Y3H assay between STX13 and AP-3 or AP-1 hemicomplexes.** Y2HGold yeast strain expressing respective bait and prey plasmids as shown in the figure (also refer Fig. 4F) were lysed and then subjected to immunoblotting. Blots were probed with anti-STX13, anti-TYR, anti-Myc or anti-HA antibodies. Note that all subunits of AP-3 or AP-1 contain HA-epitope and STX13 and TYR were probed with their respective antibodies. Expression of empty vectors was also shown separately. **(D) Y2H assay between STX13 and VAMP7, and the analysis of protein expression.** Transformed the yeast strain Y2HGold with STX13 (WT or Y3F mutant) bait and different VAMP7 prey plasmids as shown in the figure (also see Table S1). For reporter activity, selected the transformants on +His, –His and –His (2 mM 3AT) plates. Yeast transformants were lysed and then immunoblotted. Blots were probed with anti-HA or anti-Myc antibodies for VAMP7 or STX13 expression respectively.

Supplementary Movies: Respective cell types were transfected with GFP-STX13^{WT} or GFP-STX13$^{\Delta129}$ or GFP-VAMP7, imaged by live imaging fluorescence microscopy for 5 min as described in the Materials and Methods. The image series were analyzed by ImageJ software. Movies were converted into 'avi' format using ImageJ and displayed at 3 fps (frames per second).

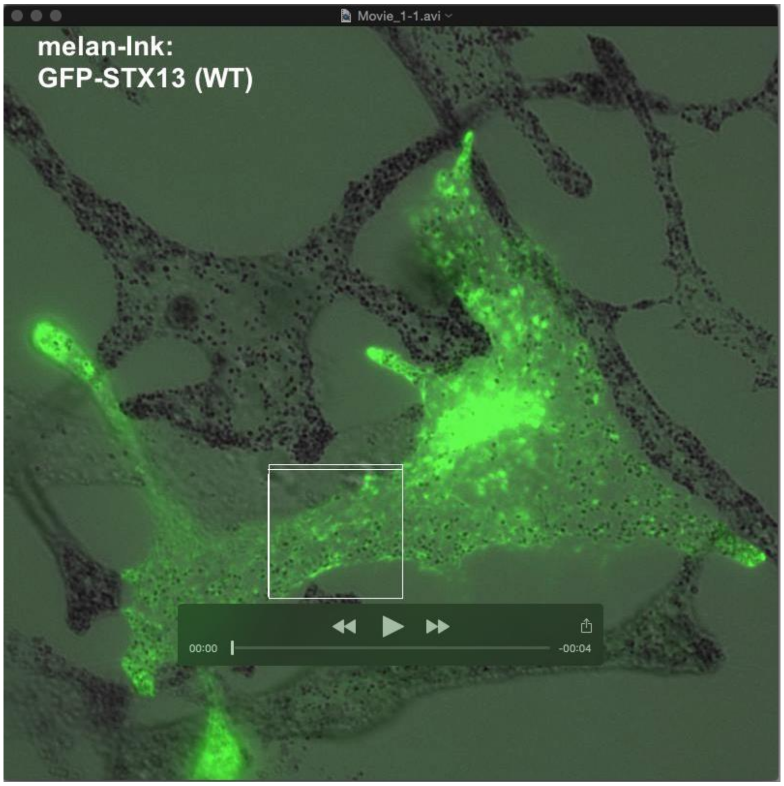

Movie 1. Time-lapse imaging of GFP-STX13^{WT} in wild-type (melan-Ink4a) melanocytes.

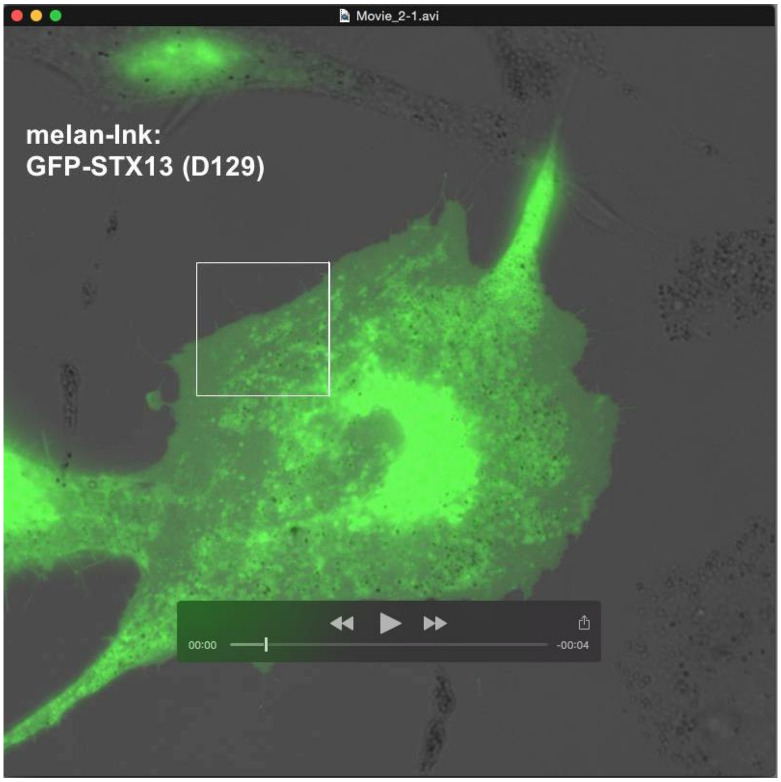

Movie 2. Time-lapse imaging of GFP-STX13$^{\Delta 129}$ in wild-type (melan-Ink4a) melanocytes.

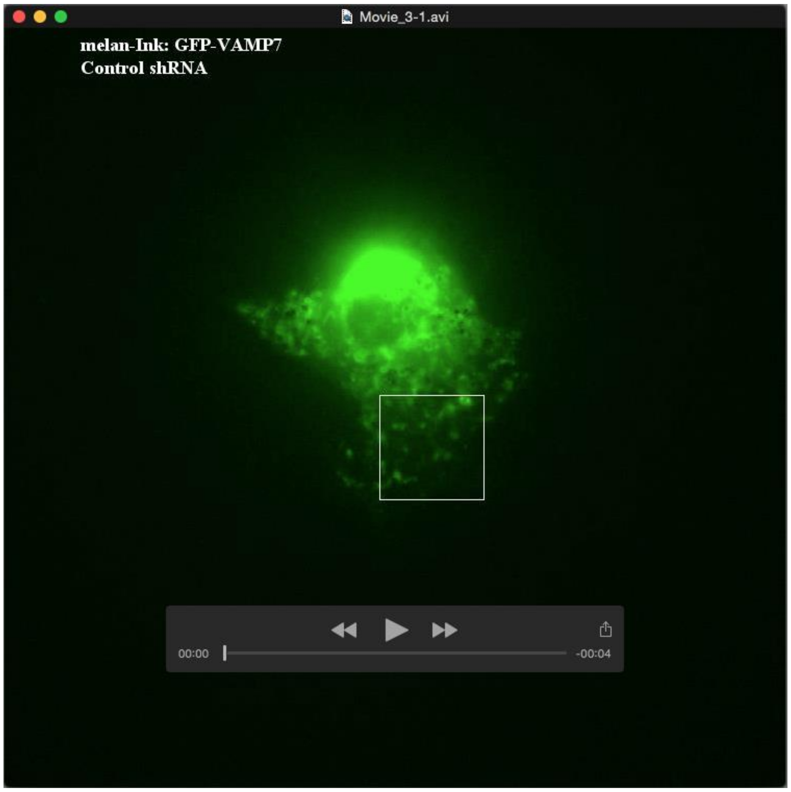

Movie 3. Video microscopy of GFP-VAMP7 in control shRNA transduced wild-type melanocytes.

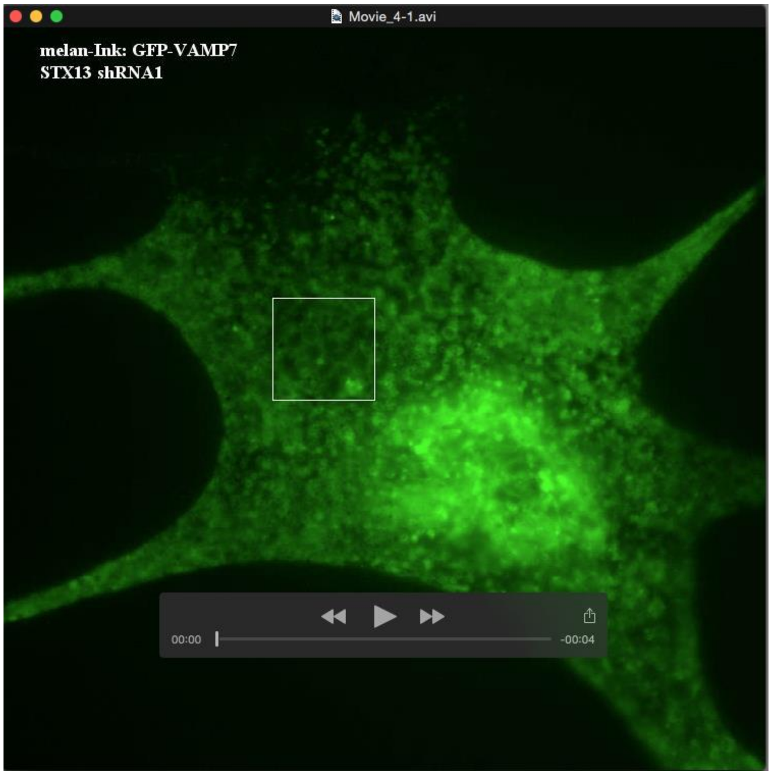

Movie 4. Video microscopy of GFP-VAMP7 in STX13 shRNA-1 transduced wild-type melanocytes.

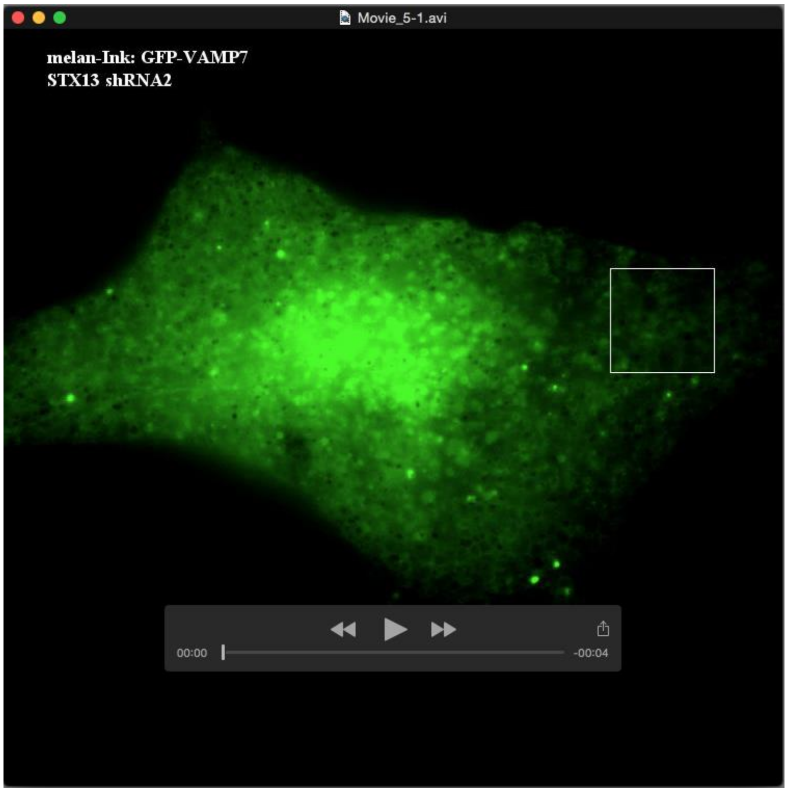

Movie 5. Video microscopy of GFP-VAMP7 in STX13 shRNA-2 transduced wild-type melanocytes.

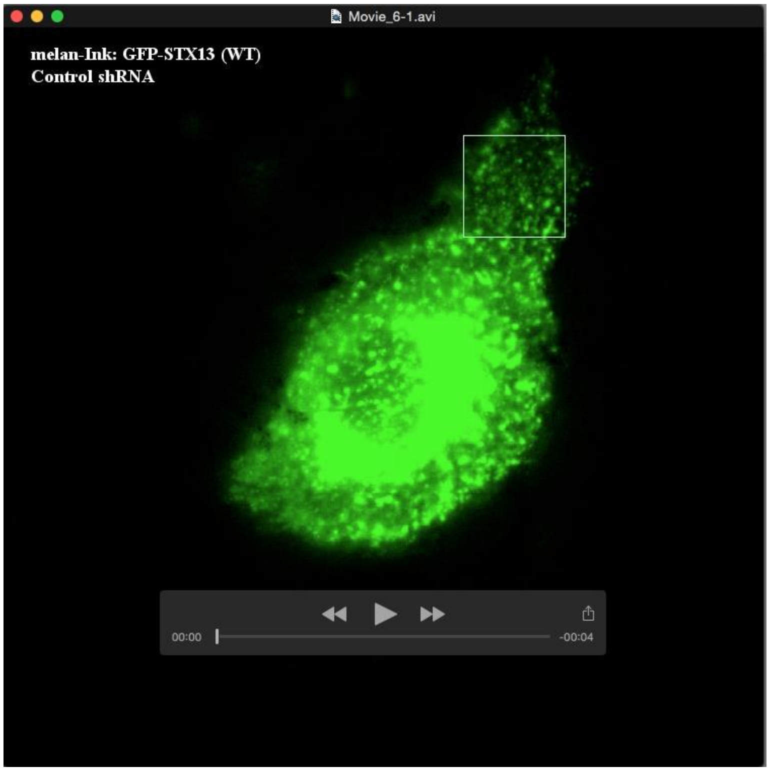

Movie 6. Video microscopy of GFP-STX13^{WT} in control shRNA transduced wild-type melanocytes.

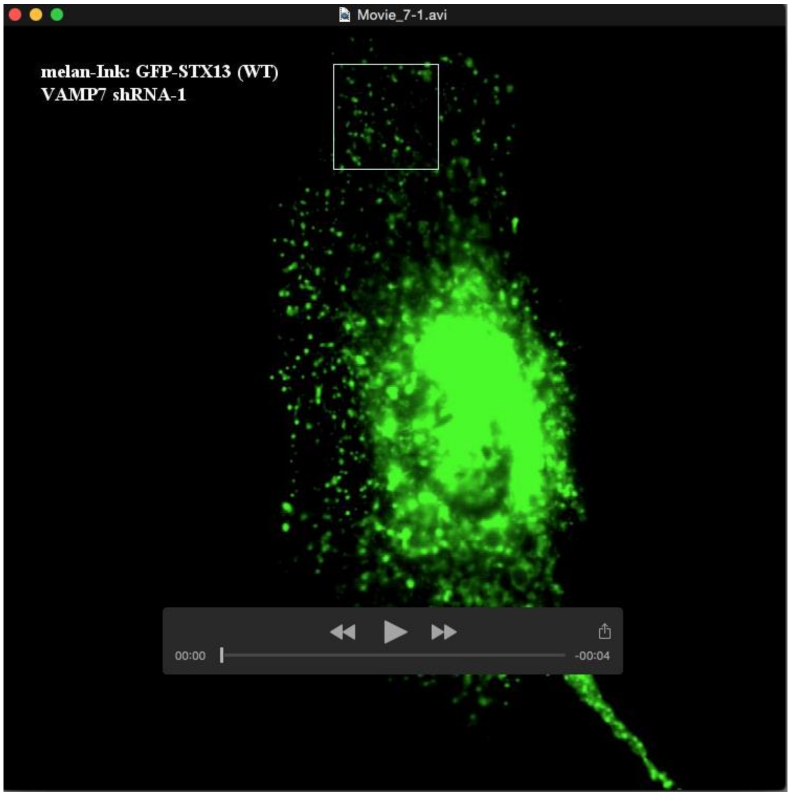

Movie 7. Video microscopy of GFP-STX13^{WT} in VAMP-7 shRNA-1 transduced wild-type melanocytes.

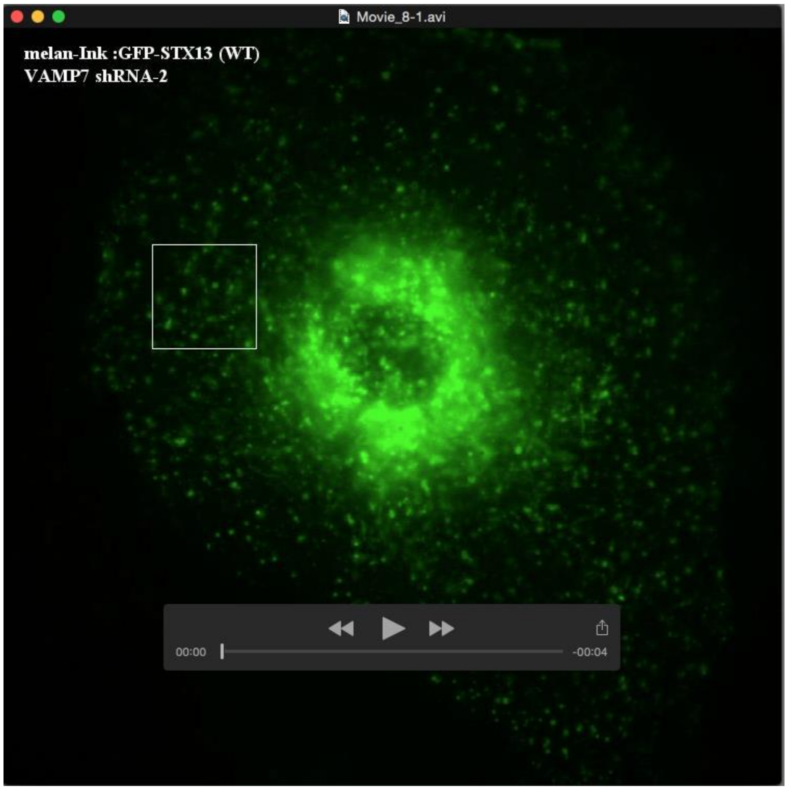

Movie 8. Video microscopy of GFP-STX13^{WT} in VAMP-7 shRNA-2 transduced wild-type melanocytes.

Supplementary References:

Chaudhuri, R., Lindwasser, O. W., Smith, W. J., Hurley, J. H. and Bonifacino, J. S. (2007). Downregulation of CD4 by human immunodeficiency virus type 1 Nef is dependent on clathrin and involves direct interaction of Nef with the AP2 clathrin adaptor. *J. Virol.* **81**, 3877-3890.

Janvier, K., Kato, Y., Boehm, M., Rose, J. R., Martina, J. A., Kim, B. Y., Venkatesan, S. and Bonifacino, J. S. (2003). Recognition of dileucine-based sorting signals from HIV-1 Nef and LIMP-II by the AP-1 gamma-sigma1 and AP-3 delta-sigma3 hemicomplexes. *J. Cell Biol.* **163**, 1281-1290.

Sitaram, A., Dennis, M. K., Chaudhuri, R., De Jesus-Rojas, W., Tenza, D., Setty, S. R., Wood, C. S., Sviderskaya, E. V., Bennett, D. C., Raposo, G. et al. (2012). Differential recognition of a dileucine-based sorting signal by AP-1 and AP-3 reveals a requirement for both BLOC-1 and AP-3 in delivery of OCA2 to melanosomes. *Mol. Biol. Cell* **23**, 3178-92.
